# Transcriptomic analysis of the tick midgut and salivary gland responses upon repeated blood-feeding on a vertebrate host

**DOI:** 10.3389/fcimb.2022.919786

**Published:** 2022-08-04

**Authors:** José María Medina, Mohamed Amine Jmel, Brent Cuveele, Cristina Gómez-Martín, Ernesto Aparicio-Puerta, Imen Mekki, Jan Kotál, Larissa Almeida Martins, Michael Hackenberg, Chaima Bensaoud, Michail Kotsyfakis

**Affiliations:** ^1^ Dpto. de Genética, Facultad de Ciencias, Universidad de Granada, Granada, Spain; ^2^ Lab. de Bioinformática, Centro de Investigación Biomédica, PTS, Instituto de Biotecnología, Granada, Spain; ^3^ Institute of Parasitology, Biology Centre, Czech Academy of Sciences, Budweis, Czechia; ^4^ Department of Pathology, Amsterdam UMC, Location Vrije Universiteit Amsterdam, Amsterdam, Netherlands; ^5^ Program Imaging and Biomarkers, Cancer Center Amsterdam, Amsterdam, Netherlands; ^6^ Chair for Clinical Bioinformatics, Saarland University, Saarbrücken, Germany; ^7^ Faculty of Science, University of South Bohemia, Ceske Budejovice, Czechia

**Keywords:** ticks, salivary glands, midgut, repeated exposure, transcriptome

## Abstract

Ticks are blood-feeding arthropods that use the components of their salivary glands to counter the host’s hemostatic, inflammatory, and immune responses. The tick midgut also plays a crucial role in hematophagy. It is responsible for managing blood meals (storage and digestion) and protecting against host immunity and pathogen infections. Previous transcriptomic studies revealed the complexity of tick sialomes (salivary gland transcriptomes) and mialomes (midgut transcriptomes) which encode for protease inhibitors, lipocalins (histamine-binding proteins), disintegrins, enzymes, and several other tick-specific proteins. Several studies have demonstrated that mammalian hosts acquire tick resistance against repeated tick bites. Consequently, there is an urgent need to uncover how tick sialomes and mialomes respond to resistant hosts, as they may serve to develop novel tick control strategies and applications. Here, we mimicked natural repeated tick bites in a laboratory setting and analyzed gene expression dynamics in the salivary glands and midguts of adult female ticks. Rabbits were subjected to a primary (feeding on a naive host) and a secondary infestation of the same host (we re-exposed the hosts but to other ticks). We used single salivary glands and midguts dissected from individual siblings adult pathogen-free female *Ixodes ricinus* to reduce genetic variability between individual ticks. The comprehensive analysis of 88 obtained RNA-seq data sets allows us to provide high-quality annotated sialomes and mialomes from individual ticks. Comparisons between fed/unfed, timepoints, and exposures yielded as many as 3000 putative differentially expressed genes (DEG). Interestingly, when classifying the exposure DEGs by means of a clustering approach we observed that the majority of these genes show increased expression at early feeding time-points in the mid-gut of re-exposed ticks. The existence of clearly defined groups of genes with highly similar responses to re-exposure suggests the existence of molecular swiches. *In silico* functional analysis shows that these early feeding reexposure response genes form a dense interaction network at protein level being related to virtually all aspects of gene expression regulation and glycosylation. The processed data is available through an easy-to-use database-associated webpage (https://arn.ugr.es/IxoriDB/) that can serve as a valuable resource for tick research.

## 1 Introduction

Ticks are obligate hematophagous arthropods, with great importance in human and veterinary medicine (Cupp, 1991; de La Fuente et al., 2008). They can transmit a wide variety of pathogens, including viruses, bacteria, and protozoans, which can cause severe deadly diseases such as Lyme borreliosis and tick-borne encephalitis (Brites-Neto et al., 2015). Tick-borne diseases have become increasingly prevalent over the last 30 years as new habitats have been colonized by ticks under climate change and human activities, including habitat changes, deforestation, globalization of the economy, international animal migration, and urbanization (Gray et al., 2009).

Hard ticks discretely and solidly attach to their hosts, penetrate the epidermis and the dermis using their chelicerae, and inject their hypostomes into the wound (Pham et al., 2021). Small blood vessels and capillaries are consequently damaged in the host, causing skin damage with hemorrhagic lesions (Aounallah et al., 2020). The prolonged contact between ticks and their hosts on the skin surface facilitates the transmission of pathogens (Kazimírová and Štibrániová, 2013). Three feeding phases characterize hard ticks’ blood intake; it starts with the attachment to the vertebrate host that can take up to a day. The second and most extended phase, also called the slow feeding phase, usually takes days during which the hard tick acquires its blood meal before finishing the feeding process with the fast-feeding phase, which usually takes one day (Tirloni et al., 2020; Jmel et al., 2021).

The impact of ticks on animal breeding is of global economic concern. *Rhipicephalus microplus* causes 2.5 billion USD in annual losses in tropical and subtropical regions (Barker and Walker, 2014). In addition, several factors such as deforestation or climate change caused the uncontrolled interactions between human and animal habitats leading to a significant increase in the cases of tick-borne diseases and an increased social concern because of the absence of knowledge about the long-term health consequences and the lack of efficient tick-control strategies (Jia et al., 2020). A recent concrete example was observed in the United States, where several states were invaded by *Haemaphysalis longicornis* (Ben Beard et al., 2019).

Despite numerous biotechnological advances, the most common anti-tick strategy remains limited to chemical acaricides despite the harmful effects on the environment and the improved resistance of ticks to these chemical methods (Gulia-Nuss et al., 2016). Thus, novel environment-friendly, anti-tick strategies should be developed based on a deeper understanding of tick behavior and physiology. For instance, the sequencing of the genomes of *Ixodes scapularis*, *I. persulcatus*, *Rh. microplus*, *Rh. sanguineus*, *Hyalomma asiaticum*, or *Dermacentor silvarum* represent crucial turning points in understanding the aforementioned ticks’ behavior and biology for the development of subsequent anti-tick strategies (Chmelař et al., 2016). Next-generation sequencing technologies have revolutionized the genomics and transcriptomics field by replacing classical Sanger sequencing with high throughput methods allowing the handling of entire genomes and deeper coverage of transcriptomes (Schwarz et al., 2013). The transcriptome analysis of various hard ticks revealed an important number of proteins coded by differentially expressed transcripts (Francischetti et al., 2005; Tan et al., 2015).

These variable transcripts were positively selected (Tirloni et al., 2020) and the dynamics in their expression are a tick mechanism of immune evasion, also known as sialome switching (Karim and Ribeiro, 2015; Perner et al., 2018; Nuttall, 2019). At the early unfed stages of the tick life cycle (eggs, embryogenesis), basic genes such as energy metabolism pathway genes are intensively expressed (Kotsarenko et al., 2020). Genes involved in parasite-host interactions are actively expressed when the tick is in feeding stages (nymphs or female adult) on a resistant host as illustrated by the study of the transcriptome and proteome of *Rh. microplus* larvae and salivary glands of male and female nymphs while feeding on a bovine host (Garcia et al., 2020).

As previously reported, the variability of tick feeding conditions directly affects the salivary gland proteome of hard ticks (Luca et al., 2010). Thus, a solid experimental design is necessary to study the transcriptomes and proteomes of ticks efficiently. We analyzed the gene expression dynamics in the salivary glands and midguts of adult female ticks upon primary (feeding on a naive host) or secondary infestation of the same host (the host was exposed to ticks for the second time). We used single salivary glands and midguts dissected from individual adult pathogen-free female *I. ricinus *from the same “mother” to reduce genetic variability between tick individuals. The data presented in this study improves the current knowledge about *I. ricinus* salivary gland transcriptomes and paves the way towards identifying potential bioactive candidates and tick control strategies.

## 2 Materials and methods

### 2.1 Experimental workflow

**Figure 1:**
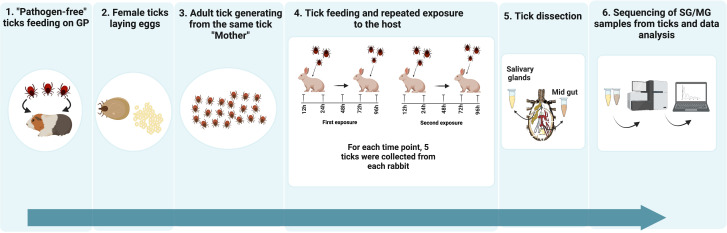
Schematic illustration of the experimental design. 1) Pathogen-free ticks feeding on GP: A generation of pathogen-free sibling ticks was hatched, fed on SPF guinea pigs and allowed to grow into adult ticks 2) Egg laying: Fully engorged female tick were fed on a SPF guinea pig until fully engorged and allowed to lay eggs. 3) New generation of adult ticks from the same tick “mother”: Molted larvae were fed on SPF guinea pigs (GP) – (ca. 100 larvae per GP and obtained adult female ticks, coming from the same “mother” were pre-mated with sibling tick males, and used for feeding and tissue isolation). The use of individuals originating from the same female tick should decrease the variability between the samples. 4) Tick feeding and repeated exposure to host: Two rabbits were exposed each to 25 female and 25 male ticks. Five females were collected at time points 12, 24, 48, 72 and 96 hours post exposure. Two weeks after the end of the first rabbit exposure to ticks, a second exposure was carried out following the same protocol. 5) Tick dissection: Ticks were dissected and their salivary glands (SGs) and midgut (MGs) were stored in 200 µL of RNAlatter at 4°C overnight followed by at -80°C. 6) Sequencing of SG/MG samples from tick individuals and data analysis: RNA was extracted from 5 SG and 3 MG samples, for each time point. Sequencing was performed using Illumina technology.

The transcriptomic study of tick salivary glands and midguts after repeated feeding on rabbit is summarized in **Figure 1**.

### 2.2 Ticks and laboratory animals

To study differentially expressed genes when *Ixodes ricinus* ticks are fed on naive or rabbits previously exposed to ticks, we hatched a generation of specific pathogen-free (SPF) sibling ticks and let them grow into adult females. The latter were fed on a SPF guinea pig until fully engorged and left to lay eggs. Molted larvae were fed on SPF guinea pigs (GP) – (ca. 100 larvae per GP and obtained adult female ticks, coming from the same “mother”, were pre-mated with sibling tick males and used for feeding and tissue isolation. The use of individuals originating from the same female tick should decrease the variability between the samples.

### 2.3 Tick feeding and repeated exposure to hosts

Two rabbits were each exposed to 25 female and 25 male ticks. Five females were collected at time points 12, 24, 48, 72, and 96 hours post-exposure. Ticks were dissected, and their salivary glands (SGs), and midguts (MGs) were stored in 200 µL of RNAlater at 4°C overnight before long-term storage at -80°C. Two weeks after the end of the first rabbit exposure to ticks; a second exposure was carried out following the same protocol. 5 SG samples from unfed ticks and from ticks fed till each feeding time point and 3 samples of MG from unfed ticks and from ticks fed till each time point were collected. Thus, a total of 55 samples from SG and 33 samples from MG were used for the sequencing protocol. Weight distribution and weight gain dynamics can be found in **Supplementary Figure 2**.

### 2.4 Sample preparation and RNA extraction

RNA from SG and MG samples was extracted with the Qiagen Tissuelyzer and the Qiagen RNA Micro Plus Kit # 74034. We used the library kits from New England Biolabs # E7490L Poly A mRNA Magnetic Isolation Module and #E7420L NebNex Ultra Directional RNA Library Prep Kit. Total RNA samples were submitted to the North Carolina State Genomic Sciences Laboratory (Raleigh, NC, USA) for Illumina RNA library construction and sequencing. Before library construction, RNA integrity, purity, and concentration were assessed using an Agilent 2100 Bioanalyzer with an RNA 6000 Nano Chip (Agilent Technologies, USA). Purification of messenger RNA (mRNA) was performed using the oligo-dT beads provided in the NEBNExt Poly(A) mRNA Magnetic Isolation Module (New England Biolabs, USA). Complementary DNA (cDNA) libraries for Illumina sequencing were constructed using the NEBNext Ultra Directional RNA Library Prep Kit (NEB) and NEBNext Mulitplex Oligos for Illumina (NEB) using the manufacturer-specified protocol. Briefly, the mRNA was chemically fragmented and primed with random oligos for first-strand cDNA synthesis. Second strand cDNA synthesis was then carried out with dUTPs to preserve strand orientation information. The double-stranded cDNA was then purified, end-repaired and “a-tailed” for adaptor ligation. Following ligation, the samples were size selected to a final library size (adapters included) of 300-450bp using AMPure XP bead isolation (Beckman Coulter, USA). Library enrichment was performed, and specific indices for each sample were added during the protocol-specified PCR amplification. The amplified library fragments were purified and checked for size and final concentration using an Agilent 2200 Tapestation (Agilent Technologies, USA).

### 2.5 Illumina sequencing

The final quantified libraries were then pooled in equimolar amounts for clustering and sequencing on a NovaSeq 6000 DNA sequencer, utilizing an S4, 150x2 paired-end sequencing reagent kit (Illumina, USA). The software package Real Time Analysis (RTA), was used to generate raw bcl, or base call files, which were then de-multiplexed by sample into fastq files for data delivery.

### 2.6 Transcriptome assembly

In order to generate a high-quality transcriptome, we first eliminated adapter sequences and low quality reads by means of Trim Galore (Babraham Bioinformatics, 2018). The preprocessed reads were then used to generate *de novo* assemblies for salivary glands (SG) and midgut (MG) separately by means of Trinity version 2.8.6 (Grabherr et al., 2011) with default parameters. To generate a consensus transcriptome, the SG and MG contigs were clustered together by applying CD-HIT (Fu et al., 2012) version 4.8.1. We considered two contigs as belonging to the same cluster if they share sequence similarity above 98% and a sequence coverage higher than 80%. Only the longest sequence within each cluster is used for further analysis. Please note that in this way, not only MG and SG assemblies become merged together, but also highly similar contigs within the individual assemblies are removed.

We analyzed several metrics of the transcriptome to ensure its good quality and completeness. The script TrinityStats.pl was used to calculate the N50 statistic and the median contig length. The completeness of the final transcriptome was assessed using BUSCO version 4.0.6 (Seppey et al., 2019), using the Arachnida lineage dataset as reference (*arachnida_odb10*). To obtain the assembly’s RNA-Seq read representation, we aligned the trimmed and quality filtered reads to our final transcriptome using Bowtie2, version 2.3.4.1 (Langmead and Salzberg, 2012) and Samtools (Li et al., 2009). To make the principal component analysis (PCA), hierarchical clustering analysis (HCA) and the correlation analysis of the expression profiles a homemade Python script was made using the libraries Scikit-learn and SciPy.

To quantify and remove the presence of contigs that presumably belong to the host, we aligned the final set of contigs to the genome of *Oryctolagus cuniculus*, version 2.0. To do so, we used STAR and STARlong (Dobin et al., 2013), version 2.7.6a, for sequences shorter than 650 pb and for sequences equal or longer than 650 pb, respectively. For the alignment to be successful, the ratio of mismatches to contig length must be 5% or lower.

### 2.7 Transcriptome annotation

Firstly, to annotate the transcriptome, we split the transcriptome into coding and non-coding transcripts. To achieve that, we obtained the candidate coding regions by means of TransDecoder version 5.5.0. Four steps were made to obtain the final set of coding regions: 1) ORFs longer than 50 amino acids were obtained using TransDecoder.LongOrfs. 2) Then, we aligned the output of TransDecoder. LongOrfs with a local database made from the proteomes of 6 organisms belonging to the taxonomic class Arachnida*: I. scapularis, Dinothrombium tinctorium, Tetranychus urticae, Tropilaelaps mercedesae, Leptotrombidium deliense and Stegodyphus mimosarum*. These proteomes were retrieved from the Uniprot database (Consortium TU, 2018). To do so Blastp version 2.10.1+ (Altschul et al., 1990) was used, with an E value cut-off of 10-5. 3) To obtain the candidate coding regions, we used TransDecoder.Predict. 4) Lastly, CD-HIT version 4.8.1 (Fu et al., 2012) was used to get the final set of unique coding regions.

Then, to get a more comprehensive knowledge about proteins that may be coded by our contigs, we extensively annotated contigs that had a coding region associated. We used Blastp version 2.10.1+ (Altschul et al., 1990) to map the unique coding regions to the following four databases: the local Arachnida database, Swiss-Prot, Uniref90, and TickSialoFam (Boutet et al., 2007; Suzek et al., 2015; Ribeiro and Mans, 2020). As recommended by Ribeiro et al., we used the E-value threshold equal to 10^-4^ for the alignment (Ribeiro and Mans, 2020). To select the best alignment, we used the Bit-score, a similarity score independent of the size of the database used. Once the best alignment was established, we used UniprotKB (Boutet et al., 2007) to find the GO annotation referring to the ID of the protein that best aligned with the unique coding region. The workflow of Gene Ontology Annotation of Blast2GO version 5.2.5 (Götz et al., 2008) was followed to annotate all those unique coding regions which did not get an annotation using UniprotKB. To obtain the conserved protein domains of our set of unique coding regions InterProScan version 5.48-83.0 (Jones et al., 2014) was used. We used Phobius (Käll et al., 2007) and SignalP version 4.1 (Almagro Armenteros et al., 2019) with sensitive parameters (-U 0.34 -u 0.34) to explore the existence of signal peptides in our set of unique coding regions and to get their putative mature peptides. TMHMM version 2.0c (Krogh et al., 2001) was used to obtain the transmembrane helices in both the set of unique coding regions and the mature peptides.

### 2.8 Expression analysis

We obtained the expression profile of the sequences from the final transcriptome by means of RSEM version 1.3.3 (Li and Dewey, 2011). This tool applies Bowtie2 to align the preprocessed reads of all 88 samples to the consensus transcriptome generating, among other statistics, the fragments per kilobase million (FPKM) expression values for each transcript. We removed lowly expressed transcripts that do not fulfill a minimum expression of 5 FPKM in all samples of at least one condition.

To make the classical differential expression analysis we used EBSeq, which is built into RSEM version 1.3.3 (Li and Dewey, 2011). We used the “expected counts” calculated by RSEM to get the differential expression for every comparison following these steps: 1) The expression of every sample was calculated using rsem-calculate-expression with the parameters –bowtie2 and –paired-end. 2) We generated a matrix for every comparison done in our classical analysis using rsem-generate-data-matrix. 3) Using this matrix and rsem-run-ebseq, the differential expression was calculated. 4) rsem-control-fdr with a 0.05 threshold was used to filter those differentially expressed genes with an FDR lower than 0.05. 5) Only transcripts with at least 200 as a mean coverage between the conditions compared remained for further analyses.

On the other hand, we used MaSigPro version 1.68.0 (Nueda et al., 2014) to make the time course analysis. An expression matrix were constructed with the expected counts provided by RSEM (Li and Dewey, 2011). Since MaSigPro does not include a normalization method, we previously normalized the expected counts using TMM normalization by means of EdgeR (Robinson and Oshlack, 2010; Robinson et al., 2010). The design matrix were made to assess differences between first and second exposure in the time course. A maximum FDR of 0.05 was requested for a gene to be considered DEG. We set the degree of the polynomial regression used for the second step of MaSigPro pipeline to 4. The forward elimination algorithm, with a minimum R squared of 0.6, was used for the stepwise regression. Lastly, 9 clusters per tissue were obtained using hierarchical clustering based on correlation distance and by means of the Ward’s agglomeration method.

To further understand the differences between ticks feeding from naïve or immunized hosts, we identified the proteins with the highest differential expression between these conditions through a pipeline based in cumulative effect calculation (Varoquaux and Purdom, 2020)We got the cumulative effect of the absolute log-fold change between first and second exposure of the host as a metric to select the most differentially expressed transcripts in the time course between both exposures of the host. This process was done for both midgut and salivary glands. The 50 DEGs with the most accumulated absolute log-fold change between the first and second exposure of the host for midgut and salivary glands were functionally *in silico* analyzed.

### 2.9 Variation analysis

Coefficients of variation were calculated for every unique coding region and condition for those samples with an expression of 5FPKM or higher in at least one of the biological replicates for the target condition. This filter is applied to avoid high coefficients of variation due to unique coding regions almost unexpressed in biological replicates of a condition. Additionally, a ranking of variation for every unique coding region for each condition in both midgut and salivary glands was calculated. Sets of 1000 unique coding regions with the highest and lowest relative variation for each condition in salivary glands were obtained and functionally analyzed *in silico*.

### 2.10 Functional *in silico* analysis

Due to the high number of conditions and comparisons made in this study, we obtained a large number of groups of interesting transcripts. To further analyze these groups, their intersections and their complex relationships, we applied SuperExactTest (Wang et al., 2015). This tool allows the user to calculate and represent intersections among multiple sets.

Once the groups and intersections of transcripts of interest were defined, we obtained their functional enrichment and protein-protein interaction networks. This was done by means of StringDB, version 11.0b (Szklarczyk et al., 2019), through the mapping to *Drosophila melanogaster.* This allowed us to get a more comprehensive knowledge about the relationship of proteins coded by transcripts, and whether these relationships configured a significant interaction network with significantly enriched associated GO terms or not.

### 2.11 Availability of data and materials

We deposited raw sequence reads in the NCBI under Bioproject accessionnumber PRJNA716261, BioSample Accession: SAMN18644895 - SAMN18644981. The Short readproject were deposited under the SRA Accession: SRR14454946 - SRR14455033. The TranscriptomeShotgun Assembly project has been deposited at https://dataview.ncbi.nlm.nih.gov/object/PRJNA716261?reviewer=lhohun1qf9keoqui6jnl31pelg.

### 2.12 Database-associated webpage - IxoriDB

IxoriDB was created using the high-level Python web framework Django, version 3.1.5 (Django Software Foundation, 2019). Django allowed us to integrate Python, HTML, CSS and JavaScript files to fully develop IxoriDB. This database-associated webpage offers the user an easy and useful way to browse the transcriptome and the features of its unique coding regions, and to gather information about other sequences provided by the user through the use of Blast, version 2.10.1+ (Altschul et al., 1990). To show the results obtained from Blast, a modified version of BlasterJS (Blanco-Míguez et al., 2018) was used. The webpage is accessible through https://arn.ugr.es/IxoriDB.

### 2.13 Confirmation and quantification of gene expression by cDNA synthesis and RT-qPCR

In order to perform the transcriptome validation we used a different batch of ticks reared the same conditions as the ones used during the sequencing. Total RNA was extracted from the salivary gland and the midgut from a tick using QIAzol and miRNeasy RNA extraction kits (QIAGEN). The RNA was eluted from the column using 20 µL of RNase-free water. The quantity and quality of the samples were evaluated using a Nanodrop Spectrophotometer (Thermo Scientific). High-quality RNA samples were used for RT reactions by using a MystiCq polyadenylation cDNA synthesis kit (Sigma-Aldrich). RT was carried out by adding 2 µL of poly(A) tailing buffer (5x), 2 µL of nuclease-free water, and 1 µL of poly(A) polymerase to 5 µL of the RNA sample. The total mix of 10 µL was incubated first for 60 minutes at 37°C followed by 5 minutes at 70°C. The mixture was kept on ice and 9 µL of Mystic cDNA Reaction mix, and 1 µL of ReadyScript Reverse Transcriptase wasadded. cDNA synthesis was carried out by incubating the mixture for 20 minutes at 42°C followed by 5 minutes at 85°C. The qPCR mix for each well was comprised of 12,5 µL of 2x Maxima SYBR green/ROX master mix (Thermo Scientific), 0,3 µM of both the forward and reverse primers and then nuclease-free water was added to reach a volume of 24 µL. In each well, 1 µL of cDNA sample was added to reach a final volume of 25 µL. The reactions were performed using QuantStudio 6 Flex machine (Applied Biosystems). The following conditions were used: preincubation (2 minutes at 50°C), initial denaturation (10 minutes at 95°C), 45 cycles of denaturation (15 seconds at 95°C), annealing (30 seconds at 60°C), and extension (30 seconds at 72°C). The specificity of the amplified product was evaluated using melting curve analysis and gel electrophoresis. The expression levels were represented by their corresponding Cycle threshold (Ct) values. To compare these values, the 2-ΔCt method was used. The difference in Ct values was measured between the protein-coding genes and the average of both housekeeping genes elongation factor (EF) and Actin.

## 3 Results

### 3.1 Transcriptome assembly

**Figure 2:**
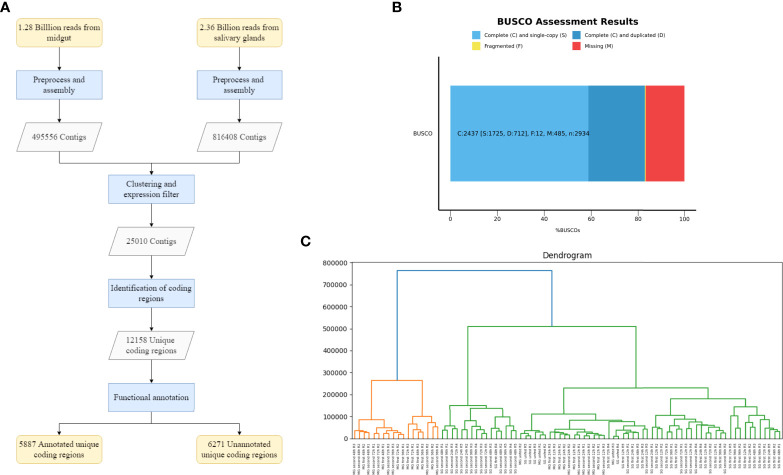
Assembly of the transcriptome and quality assessment. **(A)** Flowchart that summarizes the process and results obtained for the assembly, identification of coding regions and functional annotation of the transcriptome. **(B)** Analysis of the completeness of the transcriptome using Benchmarked Universal Single-Copy Orthologs (BUSCOs). **(C)** Hierarchical clustering of the expression pattern of the eighty-eight samples from *Ixodes ricinus*. Different clusters grouped by similarity between expression patterns are represented in this dendrogram. The Y axis represents the height of the dendrogram, the lower the height in which a clade or node appears, the more similarity between the expression patterns of the samples that belong to it. The code used for labeling samples is the following: tissue (MG or SG), exposure number (unfed, first or second), time-feeding point in hours (12h, 24h, 48h, 72h and 96h) and number of biological replica (R1, R2 or R3).

A total of 3.64 billion paired-end reads were obtained by means of high-throughput sequencing. After adapter trimming and removal of low-quality reads, 3.51 billion clean reads remained. While an *I. ricinus* genome assembly is available (Cramaro et al., 2015), it contains 204,516 contigs and therefore many genes might be either represented by several contigs or not be included at all. Therefore we decided to generate a *de novo* transcriptome assembly. In principle, two options existed: i) assembly of individual samples generating a consensus transcriptome by means of clustering approaches at a second step; ii) merging all samples together first, generating only one *de novo* assembly with Trinity. Initial exploratory analysis showed that the first option generates a much higher number of contigs, 1,770,231 contigs versus the 981,846 generated by the second option, with worse general quality parameters. Therefore, we opted to generate first one transcriptome for salivary glands and one transcriptome for midgut, separately. These primary assemblies contain 495,556 (midgut) and 816,408 (salivary glands) contigs. We then condensed the number of contigs by means of clustering highly similar sequences, obtaining a total of 981,846 contigs. The final transcriptome was obtained by applying a minimum expression filter of 5 FPKM that needs to be fulfilled by all samples of at least one condition. This final consensus transcriptome contains 25,010 contigs with a mean length of 1489 and an N50 of 2763. The transcriptome not filtered for FPKM values (981,846 contigs) had a median length of 329 and N50 of 542 showing a strong improvement in basic assembly quality statistics after filtering out lowly expressed contigs. Furthermore, we found that 87.6% of all paired-end reads can be mapped back to the assembly which is above generally observed values. As a final quality analysis of the assembly, we applied BUSCO (Seppey et al., 2019) to address the completeness of the transcriptome. Using the Arachnida lineage dataset provided by BUSCO as a reference, the analysis showed: 1) 83.1% of complete Benchmarked Universal Single-Copy Orthologs (BUSCOs), from which 24.3% were found duplicated mainly due to the existence of isoforms in the transcriptome; 2) 0.4% of fragmented BUSCOs; 3) 16.5% of missing BUSCOs (**Figure 2B**).

The next aim was to split the transcriptome into coding and non-coding transcripts. We used TransDecoder in a two-step process, obtaining a total of 13,458 coding transcripts. Given the possibility that more than one transcript codes for the same protein, we clusterized highly similar proteins. Finally, we obtained 12,158 transcripts with unique coding regions and 11,552 putatively non-coding transcripts. A summary of the workflow and primary results are presented in **Figure 2A**.

One important exploratory step consists of the detection of outlier samples. We analyzed the expression profiles of the unique coding transcripts by means of hierarchical clustering (HCA), principal component (PCA), and correlation methods. Hierarchical clustering (**Figure 2C**) shows that no single outlier samples exist but the samples form several, well defined clusters. For example, samples from the midgut of ticks fed for 48, 72 and 96 hours form clear and well-separated clusters and unfed samples show the tendency to group with early feeding time points. Furthermore, a certain separation of first and second exposure samples can be appreciated. The PCA analysis (**Supplementary Figure 1**.) confirms that no clear outlier exists. The main separation (component 1) is between midgut and salivary gland samples suggesting that the tissue is the strongest factor in determining expression values. Nevertheless, it can be observed that principal component 2 separate some samples, corresponding to biological replicates of unfed and early feeding time points, from the main cluster. The correlation matrix, ultimately supported the features displayed by the HCA, showing a strong correlation between the samples from late feeding times in midgut, even between the samples from midgut of first and second exposures. It also showed that the correlation between early feeding time point samples (12h and 24h) and late feeding time point samples (48h, 72h and 96h) is almost zero in midgut. Regarding the salivary glands, we observed a more divergent trend of correlation. (**Supplementary Figure 1**). The main conclusion of these exploratory analyses is that no outlier samples exist and therefore all samples were included in downstream analyses.

### 3.2 Transcriptome annotation

After assembly and quality assessment, the next step consisted of deeply annotating the obtained coding transcripts. We mapped the 12158 unique coding regions to four different databases: the *Arachnidae* database mentioned before, Swiss-Prot, Uniref90 and TickSialoFam (Boutet et al., 2007; Suzek et al., 2015; Ribeiro and Mans, 2020). A total of 7225 coding regions could be assigned to at least one reference sequence from one of the 4 databases. The best alignment for each coding region was selected based on the Bit-score that Blastp provides. If possible, GO terms and Keywords were assigned based on the best alignment selected using the UniprotKB (Boutet et al., 2007) database. Thus, we obtained a total of 4312 mapped and annotated coding regions. Blast2GO (Götz et al., 2008) was used to annotate the coding regions which did not align with any of the databases or did not have a GO annotation associated. Blast2GO provided an additional 1138 annotations yielding a total of 5450 annotated unique coding regions and 6708 coding transcripts that remained unannotated. Finally, we used InterProScan (Jones et al., 2014) to obtain conserved protein domain annotations and its associated GO terms if available. This provided a final set of 5887 annotated unique coding regions that have at least one assigned functional annotation from either InterPro domains or Gene Ontology. In summary, from a total of 12,158 unique coding regions, 5456 were associated with at least one GO term and under at least one GO category: 3190 (58.47%) under “biological process”, 4362 (79.95%) under “molecular function”, and 3335 (61.13%) under “cellular component”. Firstly, the majority of the unique coding regions with a biological process GO term sub-classified under “organic substance metabolic process” (2019, 63.29%), “nitrogen compound metabolic process” (1692, 53.04%), and “cellular component metabolic process” (1730, 54.23%). Secondly, regarding the unique coding regions with “molecular function” GO terms, 2648 (61.53%) were sub-classified under “binding” and 2349 (53.85%) under “catalytic activity”, with “hydrolase activity” as the most populated final node (880, 20.17%). Lastly, for the unique coding regions with “cellular component” GO terms, we found highly scored nodes as “intracellular membrane-bound organelle” (1328, 39.82%), “integral component of membrane” (1277, 38.29%) or “cytoplasm” (1378, 41.32%). The full distribution of the classification of the GO terms in our transcriptome can be found in **Supplementary Figures 3**–**5**.

We detected the presence of signal peptides and cleavage sites of the unique coding regions beginning with a methionine. The putative mature peptide was obtained for unique coding regions with a signal peptide. Additionally, we predicted the existence of putative transmembrane helices in the set of unique coding regions. This information allowed the classification of the set of unique coding regions into four different classes: 1) Putatively secreted and annotated: at least one functional annotation and a predicted signal peptide must exist but no transmembrane domain according to Ribeiro et al. (Ribeiro and Mans, 2020). 2) Putatively secreted but not annotated: this class refers to putatively secreted coding regions which do not have a GO or InterPro annotation. 3) non-secreted and annotated: this class is formed by those annotated coding regions that do not have a signal peptide, or have a signal peptide but also at least one transmembrane domain in the mature peptide. 4) non-secreted and non-annotated coding region: this class contains putatively secreted coding regions which did not have a GO or InterPro annotation. Additionally, we defined a coding region as tissue-specific if the fold-change between midgut and salivary glands is 2 or higher. The complete annotation including the classification, expression and tissue specificity can be found in **Supplementary File 1**.

### 3.3 Database-associated webpage – IxoriDB

**Figure 3:**
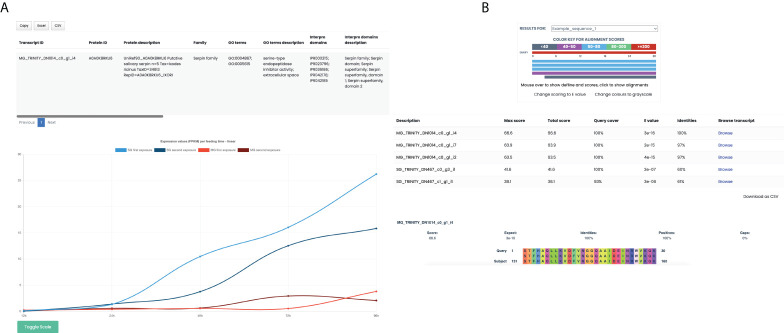
Database-associated webpage, IxoriDB. **(A)** Results offered by IxoriDB when the user browses a protein or unique coding region. The main features of the unique coding regions: Sequence, CDS, known protein that best aligns with them, functional annotation (family, GO terms and InterPro domains), putative secretion (SignalP), transmembrane domains, classification according to its annotation, and tissue specificity can be found in the table. Also, the expression among the feeding times for both midgut and salivary glands can be visualized in a graphic in which you can adjust the scale to logarithmic. **(B)** Results offered by IxoriDB when the user aligns its own sequences to our transcriptome. The information about the aligment and best representatives is shown for every sequence given by the user.

To accomplish the task of providing all the information gathered on the unique coding transcripts, we developed a query and browsable database (https://arn.ugr.es/IxoriDB). All main features like sequence, CDS, expression values homologous proteins, functional annotations (family, GO terms and InterPro domains), putative secretion (SignalP), transmembrane domains, classification according to its annotation, and tissue specificity can be easily accessed and downloaded (**Figure 3A**). The user can browse the transcriptome either by transcript name, protein name or selecting one or various options for the many available features. Additionally, the webpage allows the users to blast their own sequences to the transcriptome to get the best representative for each sequence and the features associated with it (**Figure 3B**). Lastly, the transcriptome, the CDS sequences, and an Excel sheet with all the features about every unique coding region are available in the section “Downloads” of the webpage.

### 3.4 Variation analysis

The variation of the expression of a transcript across the biological or technical replicates is important for the researchers to get an idea about the consistency, reliability and reproducibility of the results found in downstream analyses. Furthermore, for the first time we use genetic material from individual ticks here and not pools from several individuals. Therefore, individual response from the ticks to the feeding threads can manifest itself by a high fluctuation of certain genes, i.e. up or downregulation in some ticks of the same condition. To detect genes with a putative strong individual component, we calculated the coefficient of variation for every unique coding transcript in every condition and tissue. This coefficient is independent of the expression value as it normalizes the variance with the mean value. Nevertheless, extremely low expressed transcripts can obtain high CV only by chance and therefore only transcripts with at least 5 FPKM in one sample of a certain condition were considered. Additionally, a mean coefficient of variation and a variation ranking is provided for every coding transcript (**Supplementary File 2**.)

**Figure 4:**
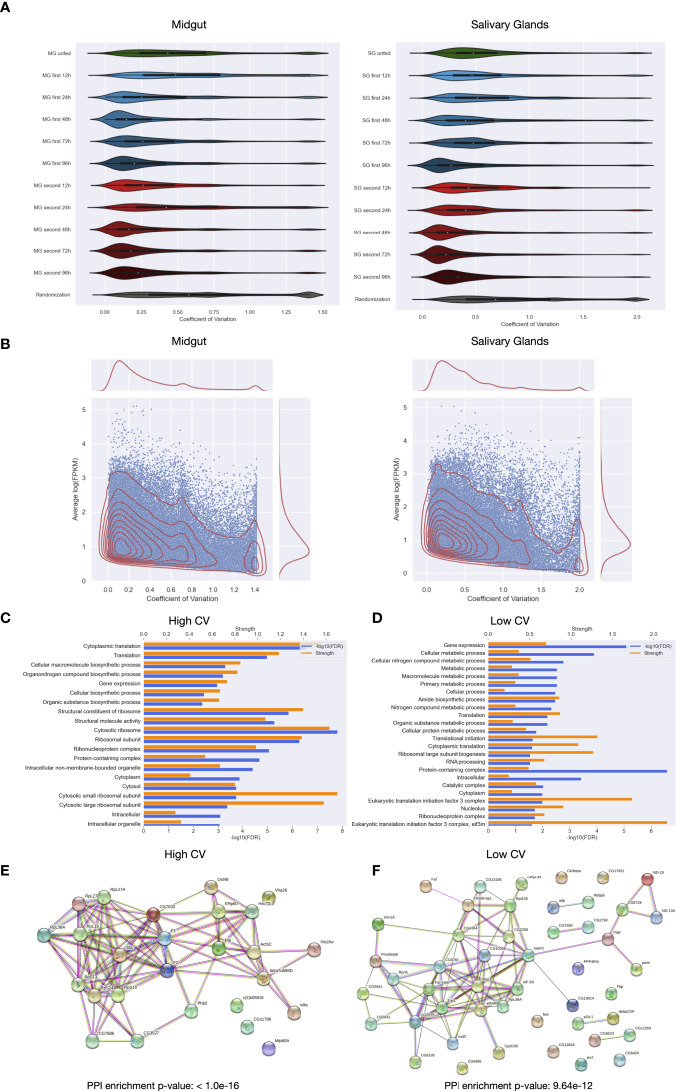
Distribution of the coefficients of variation for the samples of *Ixodes ricinus*. **(A)** Violin plots showing the distribution of coefficients of variation of unique coding regions for biological replicas per condition in midgut and salivary glands. The code used for labeling samples is the following: tissue (MG or SG), exposure number (unfed, first or second), feeding time point in hours (12h, 24h, 48h, 72h and 96h). As a negative control, a distribution of coefficients of variation taking random values for both tissues sepparately is shown in each plot. **(B)** Distribution of the coefficients of variation versus the logarithm of the mean expression of the unique coding regions in each condition for both midgut and salivary glands. **(C)** Significantly enriched GO terms in the set of unique coding regions with the highest coefficients of variation in at least eight conditions, significance and strength, which correspond to the log10(observed/expected), for each GO term are shown. **(D)** Significantly enriched GO terms in the set of unique coding regions with the lowest coefficients of variation in at least eight conditions. **(E)** Interaction network of the set of unique coding regions with the highest coefficients of variation in at least eight conditions. **(F)** Interaction network of the set of unique coding regions with the lowest coefficients of variation in at least eight conditions.

The distribution of the coefficients of variation are shown in **Figure 4A** for each condition. In order to compare the distribution with a random expectation, we generated another distribution by calculating the CVs by randomly picking 5 samples for each transcript (bottom of **Figure 4A**) out of all midgut and salivary gland samples, respectively. In general, for each condition we observed lower relative variation than what can be expected by chance. This result proves that the feeding time point and/or exposure have an impact on expression values. More interestingly is that later feeding time points show lower relative variation in both tissues. This could indicate that at the beginning of feeding, gene expression varies between individuals and towards the end the gene expression programs become more similar among the ticks. When comparing the first exposure with the second exposure of the host to ticks, lesser relative variation can be found in the second exposure, especially in salivary glands. This agrees with what we observed in the PCA analysis: some biological replicates of unfed and early feeding time points were separated from the main cluster by means of the principal component 2.

**Figure 4B** displays coefficients of variation as a function of expression. A general tendency of decreasing CV with increasing expression values can be appreciated. However, a group of highly expressed coding transcripts with high relative variation is present as well (peak on the right side of the graphic). **Figures 4C, D** show the breakdown into first and second exposure. Both are qualitatively very similar with a small decrease of highly expressed and highly fluctuating coding transcripts in the second exposure.

If the detected high fluctuation of a coding transcript in a given condition really reflects an individual component, and thus the possibility of being responding to the feeding thread, these coding transcripts should show higher dispersion in the other feeding time points or at first and second exposure as well. To test this hypothesis, we extracted sets of 1000 coding transcripts with the highest coefficients of variation for each condition in salivary glands and intersected them with each other. Thus, we obtained a set of 3358 coding transcripts having high variance in at least 8 of the 11 conditions. In general, the number of intersected coding transcripts per pair of conditions is higher that what can be expected by chance, which seems to indicate that a certain number of coding transcritps show high fluctuation in nearly all conditions. Additionally, we compared these coding transcripts with a strong individual component with the coding transcripts that shows the opposite, i. e. coding transcripts with low variance across the biological replicates of all conditions. A lesser individual component may indicate that these coding transcripts do not respond to the feeding thread, or that they respond to it without an individual component. We obtained a total of 4182 different coding transcripts having low variance in at least 8 of the 11 conditions analyzed. As the coding transcripts with high variance, the intersection per pair of conditions of coding transcripts with low variance was more than what can be expected by chance. This suggests that both set of coding transcripts with consistent low and high variation among conditions are significative, since they tend to be the same for every condition.

To elucidate this, and to learn more about the individual components of our analysis, we functionally characterized coding transcripts with high and low individual component. Firstly, we analyzed the enrichment of Gene Ontology, which showed that high variation could be found among coding transcripts with associated GO terms of vital functions such as “translation”, “structural constituent of ribosome”, “protein folding”, “gene expression”, etc. Also, functions related to biosynthetic process could be found enriched in the coding transcripts with high variance among every condition, i. e. “organonitrogen compound biosynthetic process”, “cellular macromolecule biosynthetic process” among others (**Figure 4C**). Even though we found some similarities between the enriched GO terms obtained for coding transcripts with low variance and those obtained for coding transcripts with high variance i.e “translation” or “gene expression”, we found that a lot of metabolic processes may have a low individual component (**Figure 4D**).

Then, we obtained the protein-protein interaction network of the proteins coded by transcripts with high and low variation across biological replicates, separatedly. (**Figures 4E, F**). Interaction network for coding transcripts with a strong individual component contained more interactions than what can be expected by chance and reflects what we obtained analyzing the enrichment of Gene Ontology with a high number of ribosomal proteins among other important proteins related to extremely important processes as the translation. On the other hand, the interaction network for coding transcripts with low individual component also had more interactions than what can be expected by chance. This network also showed the loss of ribosomal proteins and elongation factors with respect to the network of coding transcripts with high variation.

The same analysis was carried out for the midgut, but no significant results were obtained. To elucidate if this absence of significant results is due to lower statistical power, we compared the results obtained from midgut to those obtained mimicking the analysis with three random salivary glands samples. Results for coding transcripts with high variation are virtually maintained, but not for those with low variation, indicating that statistical power has an influence but also that differences between salivary glands and midgut exist. (**Supplementary Figure 6**).

### 3.5 Differential expression analysis

In order to elucidate the impact of the feeding time or first vs second exposure on gene expression, we carried out two types of analysis: 1) pairwise comparisons, i.e. comparing two conditions (for example unfed vs. feeding time point) and 2) global or time course analysis. The time course analysis will detect those genes that show significant changes in their expression profile between first and second exposure as feeding progresses.

#### 3.5.1 Pairwise analysis

**Figure 5:**
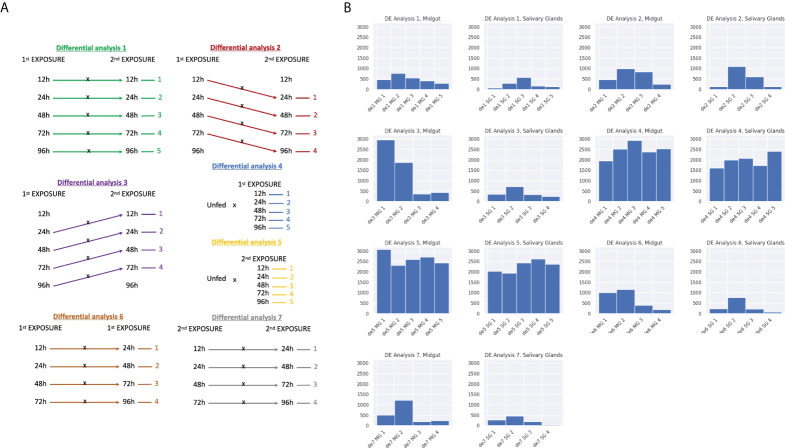
Classical differential expression analysis. **(A)** Scheme representing every comparison made for the classical differential expression analysis. Each comparison was made for both midgut and salivary glands. **(B)** The number of DEGs for every comparison is shown in these histograms. The coding for the differential analyses corresponds to those explained in Figure 5a. (B.1) *Ixodes ricinus* ticks that infected the host for the first time and were fed until 5 different time points versus *Ixodes ricinus* ticks that infected the host in a second exposure and were fed until the same time points. (B.2) Ticks that infected the host for the first time and were fed until 4 different time points versus ticks that infected the host in a second exposure and were fed until the next time point in the timeline. (B.3) Ticks that infected the host for the first time and were fed until 4 different time points versus ticks that infected the host in a second exposure and were fed until the previous time point in the timeline. (B.4) Unfed ticks against ticks that infected the host for the first time and were fed until 5 different time points. (B.5) Unfed ticks against ticks that infected the host for the second time and were fed until 5 different time points. (B.6) Ticks that infected the host for the first time versus ticks that infected the host for the first time and were fed until the next time point in the timeline. (B.7) Ticks that infected the host for the second time versus ticks that infected the host for the first time and were fed until the next time point in the timeline.

**Figure 6:**
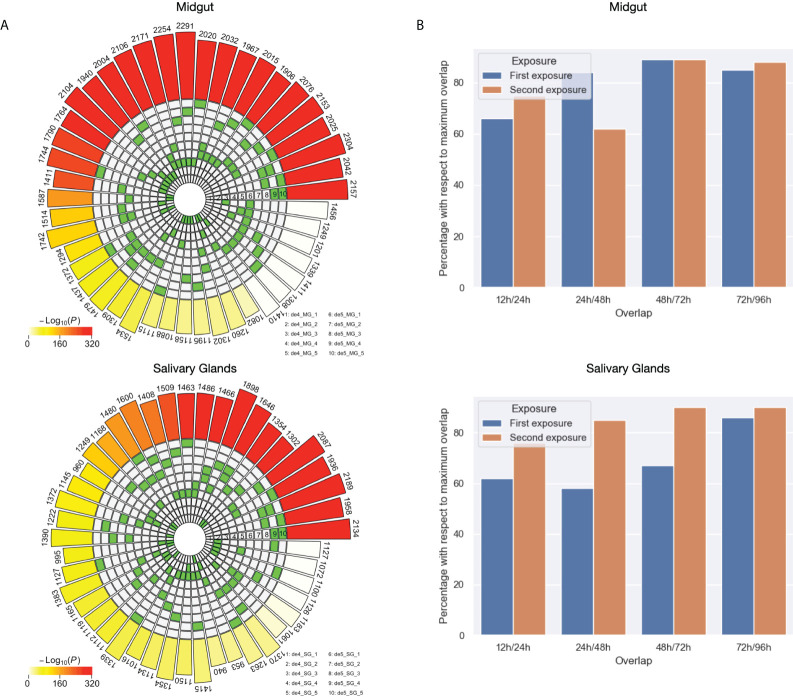
Exhaustive analysis of the differences and similarities between the DEGs against unfed in the first and second exposure to the host. **(A)** Intersection analysis for the DEGs against unfed for each condition of first and second exposure for both midgut and salivary glands. **(B)** Differences between the set of DEGs against unfed across the timeline in first and second exposures for both midgut and salivary glands. The Y axis represent percentages with respect to the maximum possible overlap of shared genes. The maximum possible overlap correspond to the size of the set of DEGs against unfed with the lowest size for each comparison.

The classical or pairwise analysis provides information about the differences between a given pair of condition. It allows us to get a global vision of the differences between ticks feeding at different time points, ticks feeding from naïve or immunized hosts or even to get some knowledge about the dynamics occurring during the time course through the intersection of sets of differentially expressed genes (DEGs). Our experimental design allows for a high number of comparisons that are summarized in **Figure 5A** (Li and Dewey, 2011).The magnitude, i.e. number of DEGs obtained for each comparison is summarized in **Figure 6B**. The highest number of DEGs can be observed for the comparisons between the different feeding time points with the unfed, both for first and second exposure. When comparing adjacent time points (DE analyses 6 and 7), a much lower number of DEGs are observed (**Figure 5B**, bottom and bottom right). Interestingly, there is common pattern shared in midgut and salivary glands that can be observed in both first and second exposures. In all 4 comparisons, 24h vs. 48h yielded the highest number of DEGs and generally the molecular differences between two adjacent time points decreased strongly at later feeding time-points.

When comparing first vs. second exposure, either at identical time points (i.e. 12h first vs. 12h second), delayed (for example, 12h first vs. 24 hours second) or advanced (for example 24h first vs. 12h second), again, a generally lower number of DEGs were obtained (lower than the numbers obtained when comparing to unfed). There seemed to be one clear exception to this general behavior we observed while testing the hypothesis that feeding is delayed in the second exposure. Nearly 3000 DEGs were obtained comparing 24h first with 12h second exposure and suntil aprox. 1900 DEGs were identified in 48h first vs. 24h second. This suggests that indeed, differences in gene regulation might exist between the first and second exposure.

Then, we calculated the intersection of the sets of DEGs against unfed ticks with each other by tissue in order to explore which feeding times are most similar and which ones differ the most. **Figure 6A** shows that towards later feeding time points, the overlap between DE genes increases. This in turn suggests less regulation of gene expression towards the end of the feeding process in both tissues, midgut and salivary glands.

Lastly, we focused on quantifying the changes in differential expression between feeding time points. **Figure 6B** depicts the normalized overlap between two adjacent time points, i.e. 100% would indicate that both sets are identical. Differences between the first and second exposure can be appreciated especially at early time points, but not towards the end of the feeding process which is in line with what we observed before. Interestingly, in the midgut, most changes in the first exposure occurred between 12 and 24 hours while in the second exposure this can be seen between 24 and 48 hours. In salivary glands, we observed generally less dynamics in the second exposure (high overlap between different adjacent time points) compared to the first exposure.

#### 3.5.2 Time course analysis

**Figure 7:**
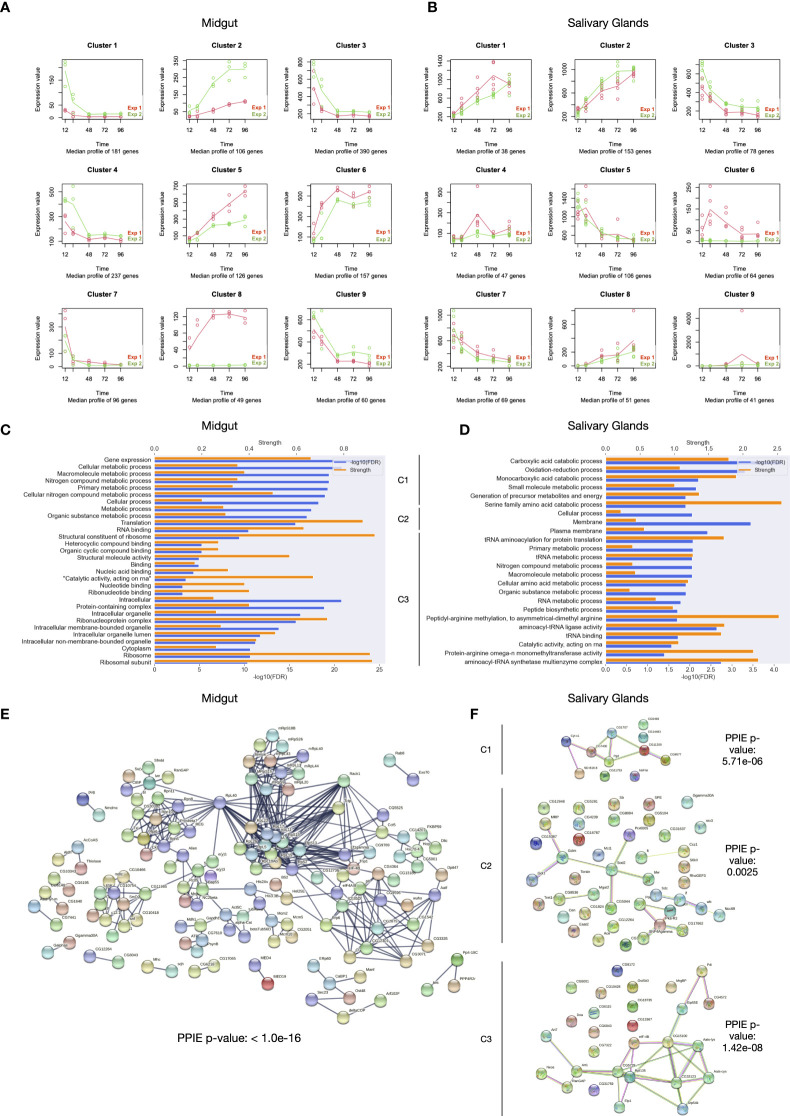
Differential expression analysis in the time course. **(A)** Clusters obtained by means of hierarchical clustering analysis based on the expression patterns of DEGs in the time course for both exposures in midgut. **(B)** Clusters obtained by means of hierarchical clustering analysis based on the expression patterns of DEGs in the time course for both exposures in salivary glands. **(C)** Ontology enrichment for the cluster coming from merging cluster 1, 3 and 4 in midgut. Significance and strength, which correspond to the log10(observed/expected), for each GO term are shown. **(D)** Ontology enrichment for the clusters 1, 2 and 3 in salivary glands. **(E)** Protein-protein interaction network for the cluster coming from merging cluster 1, 3 and 4 in midgut. **(F)** Protein-protein interaction network for the clusters 1, 2 and 3 in salivary glands.

One of the main objectives of this study was to assess the molecular response in salivary glands and midgut of the ticks upon second exposure. Differences in the expression pattern can elucidate the modulation of *Ixodes ricinus* sialome and mialome when it is invading an immunized host. For this purpose, a global time-course analysis focusing differences between first and second exposure was carried out. A total of 1402 and 647 DEGs in the time course between first and second exposure were obtained for midgut and salivary glands, respectively. We made an intersection for these sets of DEGs in the time-course, finding that 123 DEGs were common for midgut and salivary glands. The sets of time-course DEGs were then submitted to a hierarchical clustering according to their expression pattern in first and second exposure of the host. Thus, we obtained 9 clusters for midgut and salivary glands with time-course DEGs that show similar changes during the feeding time in both exposures. (**Figures 7A, B**). Distribution of DEGs over these clusters can be found in **Supplementary File 3**.

Regarding the clusters obtained taking into account differences in midgut, clusters 1, 3 and 4 represent 808 DEGs that are higher expressed in the second exposure in early stages of feeding; cluster 2 contains 106 DEGs with higher expression in late feeding points of second exposure; as the opposite, 126 DEGS with higher expression in late feeding points of first exposure belong to cluster 5; cluster 8 is formed by 49 DEGs expressed exclusively in the first exposure of the host. On the other hand, with respect to the salivary glands, cluster 1 shows 38 DEGs that are more expressed in first exposure, with the highest difference at 72 hours of feeding; cluster 2 and 3 represent 153 and 78 DEGs more expressed in the second exposure at late feeding time points and early feeding time points, respectively; cluster 4 consists in 47 DEGs that are more expressed in the first exposure at 48 hours of feeding; lastly, cluster 6, as cluster 8 for midgut, represent 64 DEGs exclusively expressed in the first exposure of the host.

To get a more comprehensive knowledge of these changes in the expression patterns during the feeding time and the differences between the first and second exposure of the host, we functionally characterized the clusters mentioned before.

In the midgut, the majority of DEGs in the time course (57.6%) are overexpressed in the second exposure at early feeding time points. Clusters 1, 3, and 4 together constituted a significant protein-protein interaction network with several GO terms enriched. Here, we found that proteins involved in vital and general processes such as gene expression, translation, or metabolic processes, among others, are overexpressed in the early stages of feeding for ticks infecting immunized hosts (**Figure 7C**). **Figure 7C** only shows the top 10 most statistically significant GO terms for biological process, cellular component and molecular function categories, respectively, the remaining GO terms are provided in **Supplementary File 4**. The protein-protein interaction network reflected this with an elevated presence of ribosomal proteins, elongation factors, proteins involved in several metabolic processes, etc (**Figure 7D**). On the other hand, we couldn’t find either significant interaction networks or significant enriched GO terms for the other interesting clusters mentioned before.

Regarding the salivary glands, even though there were significantly fewer DEGs than in the midgut, we found that clusters 1, 2, and 3 showed significant ontology enrichment and significant protein-protein interaction networks. In cluster 1 we can find that proteins involved in some metabolic processes are overexpressed in the first exposure of the host. By means of the interaction network, we had a deeper look in the components of the cluster that significantly interact with each other, finding overexpression of the delta5-delta2,4-dienoyl-CoA isomerase, which is involved in fatty acid oxidation and related to proteins involved in the mitochondrial electron transport. The interaction network created by the proteins in cluster 2 consisted of various interacting subgroups. We identified a protein involved in the behavioral and cellular response to starvation, SNF4/AMP-activated protein kinase gamma, linked to proteins that regulate neuronal activity and circadian rhythms. We found that some proteins related to ATP synthesis, detoxification activity, and cellular redox homeostasis are overexpressed in ticks in the second exposure of the host. Here, we also identified the protein alpha1,6-mannose beta1,2-N-acetylglucosaminyltransferase. This protein participates in the pathway of N-linked glycosylation, a pathway involved in the tick-host interaction. Salivary gland cluster 3 genes show a similar expression pattern and functions than those in mid gut clusters 1, 3, and 4 (**Figures 7E, F**)

**Figure 8:**
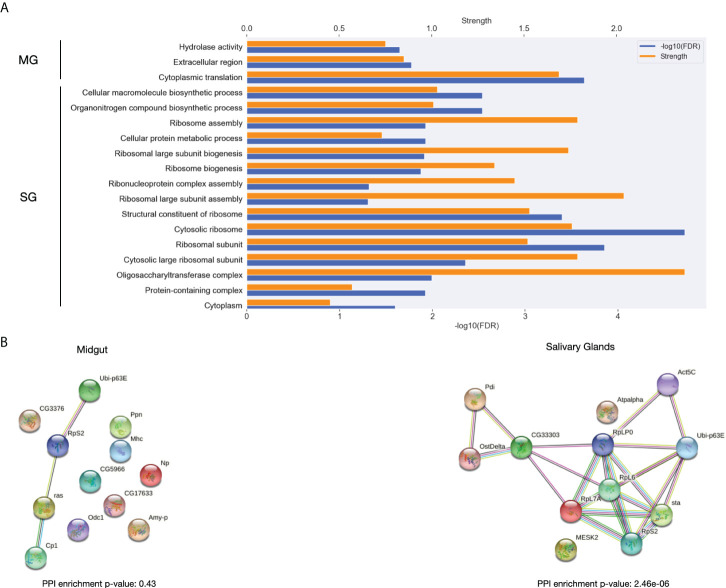
Functional in silico analysis of DEGs in the time course with the most cumulative effect of expression differences between the first and second exposure of the host during the feeding. **(A)** The most significantly enriched GO terms in the set of genes showing thedifferential expression with the most cumulative effect during the time course between first and second exposures for both midgut and salivary glands. Significance and strength, which correspond to the log10(observed/expected), for each GO term are shown. **(B)** Interaction network for the differentially expressed genes with the most cumulative effect for *Ixodes ricinus* for both midgut and salivary glands.

To further understand the differences between ticks feeding on naïve or immunized hosts, we identified the proteins with the highest differential expression between these conditions. The top 50 DEGs in the time course with the most cumulative effect of differences between first and second exposure of the host from the midgut and salivary glands were functionally characterized (**Figure 8**).

The Gene Ontology enrichment revealed that hydrolase activity, cytoplasmic translation are different between exposures in midgut. Interestingly, structural constituents of ribosome proteins were enriched among the most globally differentially expressed proteins between exposures in salivary glands. Some biosynthetic pathways were also found enriched in the DEGs with the most differences between their expression in ticks feeding from naïve and immunized host, respectively. Additionally, we found that, with a great strength of enrichment, oligosaccharil complex is significantly enriched in these DEGs.

A significantly enriched protein-protein interaction network was obtained for salivary glands but not for midgut (**Figure 8B**). Interestingly, some proteins with a role in the parasite-host interface can be found among the proteins forming the interaction network in salivary glands. In agreement with what we saw in the GO term enrichment analysis, in this interaction network we identified the subunit 1 and 2 of the dolichyl-diphosphooligosaccharide–protein glycotransferase, a crucial protein in the pathway of N-linked glycosylation (Burda and Aebi, 1999). Also, the protein disulfide isomerase (Pdi), whose N-glycosylation has been linked to virulence and to the correct folding of newly synthetized proteins in the endoplasmic reticulum was present in the network (Marín-Menguiano et al., 2019). Additionally, several ribosome proteins, a precursor of the polyubiquitin and the actin-5C was found in the interaction network.

## 4 Validation of transcriptomic data

To validate the transcriptomic data, different highly expressed protein-coding genes were selected. These genes were expected to be transcribed both in the salivary gland and in the midgut. The expected gene expression for the selected protein-coding genes from the transcriptomic data are represented by their FPKM values at different time points for both salivary glands and midgut after the first and second exposure (**Supplementary Figure 7**). To support the expected gene expression data, RT-qPCR was performed with cDNA samples that are reverse transcribed from extracted RNA samples. We have selected first and second exposure samples with a 24-hour interval to visualize changes in the expression levels. We have confirmed that the selected protein-coding genes are expressed in most samples from different time points originating from the salivary gland and midgut in both the first and second exposure. The transcripts are represented by their relative expression normalized to the average of two housekeeping genes (*ef* and *actin*) (**Supplementary Figure 8**).

## 5 Discussion

Here, we profiled transcription in the salivary glands and midguts of *I. ricinus* ticks throughout the first four days of an initial feeding and the same days of feeding on re-exposed rabbits. The novelty of our study lies in its robust experimental design, which included RNA-seq of individual pathogen-free tick tissues rather than pooled samples. The generated transcriptomes also originated from related ticks from the same “mother” to focus on experimental variables and exclude tick diversity/polymorphism (Karim and Ribeiro, 2015). Thus, our approach and the in-depth analyses provide all the advantages of biological replicates by verifying reproducibility in individual transcriptomes. It also ensures that highly, weakly, and differentially expressed transcripts are not the result of differences between individuals but rather the result of the experimental conditions (e.g., the number of exposures) and parameters (e.g., time). Transcriptomes from the same tissues, similar time points, and the same exposures tended to cluster together (**Figure 3C**). Analysis and compilation of the coefficients of variation of each transcript’s expression among biological replicates revealed that the individual transcriptomes in each group largely corresponded with each other and were stable (**Figure 4A**), ensuring that we could proceed and make valid and reliable comparisons between groups. Additionally, we fed experimental ticks on the same pair of rabbits for the first and second exposures instead of on different rabbits.

First, we performed exploratory analyses to test the degree of variation between the biological replicates. This analysis provides information about the reliability and reproducibility of the data obtained from individual ticks. Importantly, it addresses the caveats of data from pooled samples that can be skewed by outliers that nullify or exaggerate any measured biological effect. Here, we found that our data had less variation than what would be expected by chance and that the inter-replicate variation further diminishes in late feeding times points. This suggests that the completion of feeding depends on a universal genetic program or shutting off of such a program. In almost every condition, we found that the number of transcripts with high or low variation was significantly higher than would be expected by chance, which led us to functionally characterize them. We found that catabolic processes, which are associated with blood feeding and digestion [reference], are stably expressed between biological replicates. In contrast, ribosomal proteins varied strongly which indicates functional specialization and diversification as a result of general or specific regulation (Marín-Menguiano et al., 2019).

As hosts mount an immune response against tick products and can gain resistance (Francischetti et al., 2009; Perner et al., 2018), our experiment allowed us to study the capacity of ticks to counter the host immune response and adapt to feeding on previously exposed vertebrate hosts. Together, these results support existing data on tick sialome switching and reveal how ticks adapt to an immune host, which has implications for anti-tick or anti-tick-transmitted pathogen therapy.

Our results support those from existing tick transcriptomes (Karim and Ribeiro, 2015; Kotsyfakis et al., 2015) and proteomes that demonstrate that ticks can modulate their sialomes during tick feeding. This modulation, also known as sialome switch, enables ticks to evade host hemostasis and the immune system, and to complete their life cycles (Tirloni et al., 2020). Differential analyses 4, 5, 6, and 7 all demonstrate differential gene expression between time points (**Figure 6**) and indicate that sialome switching is indeed happening.

Feeding in hard ticks can be roughly divided into phases that correspond to the different blood-feeding rates and tick weight gain (Kemp et al., 1982). In parallel, the tick transcriptome changes either in preparation or due to tick feeding. Potentially, this process occurs *via* epigenetic modifications (Kotsyfakis et al., 2015) and/or signal transduction that eventually change the expression/activity of specific transcription factors (Tirloni et al., 2020). Thus, molecules such as proteases and protease inhibitors are expressed and perturb host hemostasis and immunosuppress the host (Tirloni et al., 2020). Our study also captured some of these changes in gene expression, specifically between the attachment phase and part of the slow feeding phase of tick feeding. Regardless of the exposure and the host’s immune status, the tick suntil relies on sialome switching for progression through different stages of blood-feeding. Our transcriptome analysis indicates that the most crucial switch occurred in the tick midgut between the 12- and 24-hour time points of feeding during the first exposure. This may be delayed to the period between 24 and 48 hours in the second exposure.

Ticks are ubiquitous, and each additional tick bite is a chance for pathogens to be transmitted. To interrupt both initial and subsequent tick bites, we need to understand how the tick achieves these at the molecular/transcriptomic level. Host permissibility to tick feeding varies with each tick-host interaction: tick mechanisms of evasion and whether the host is a reservoir (e.g., mice), or a non-reservoir host (e.g., humans) who can resist repeated tick feeding (Narasimhan et al., 2019). Here, we studied the rabbit that is relatively (Narasimhan et al., 2007) and at least partially resistant, permitting *I. ricinus* feeding for at least four days without ticks detaching and without a delay in tick feeding/weight gain (**Supplementary Figure 2**). Our findings and the methodology we used will help us understand how ticks can nonetheless complete a blood meal despite the host having experienced a first bite and despite evidence that they generate an adaptive immune response from the initial lesion (Trager, 1939). To directly study the host response and any additional changes in the host, we would need to distinguish the primary and secondary host responses. However, it is inherently difficult to study the proteome and transcriptome of mammalian host cells in proximity/recruited to the wound in a kinetic manner through repeated sampling, in the absence of tick molecules, and in relevant immune compartments such as the regional/draining lymph nodes responsible for generating the antibody responses. Short of generating a true host-parasite interactome, the tick transcriptomes presented here can nonetheless serve as bases to form new hypotheses on the matter, to study specific differentially expressed tick genes in the context of the host response.

In the adaptive immune response to the lesion and tick salivary secretions, we expected that the host’s accelerated, pre-emptive, and stronger response to the tick bite would also result in an accelerated response from the ectoparasite. Differential analyses 4 and 5 support this hypothesis by demonstrating that, in comparison to baseline measurements (the control “unfed” condition), we observed more differentially expressed transcripts and measured an earlier peak during the second exposure (**Figure 6B**). However, the differential gene expression is not just a question of kinetics. This is most evident in the midgut from differential analysis 3, which tests if exposure one lags behind the second exposure by one timepoint (**Figure 6B**). In a scenario in which there is such a shift, we would have observed little differential expression between e.g., the 24-hour time point of the first exposure and the 12-hour timepoint of the second exposure. However, we identified nearly 3,000 transcripts differentially expressed in this comparison. Taken together, we can hypothesize that ticks react to a previously exposed host by accelerated sialome switching (earlier initiation of a genetic program). Perhaps, these transcripts are differentially expressed to counter host molecules or host responses that are absent, delayed, or weak during the first exposure.

Time course analysis based on the differences in expression dynamics during the feeding between first and second exposure can reveal detail the tick’s response upon reexposure. This analysis showed that a greater number of DEGs in the time course with similar expression patterns can be found in the midgut rather than in salivary glands. Relatively few DEGs were shared between both tissues, suggesting that the response to the second exposure of the host is tissue-specific. In the midgut, for most genes, differences in expression dynamics between the first and second exposures were a result of accelerated early overexpression during the second exposure, in agreement with what we observed in the pairwise analysis (**Figure 5B**, DE Analyses 1 and 3). DEGs in the time course in midgut coded mostly proteins related to basic processes such as gene expression, translation or metabolism. We found a high number of ribosomal proteins among these DEGs. Regarding the salivary glands, cluster 3 showed similar expression patterns and functions to the predominant DEGs in midgut. In cluster 1, which was constituted by DEGs overexpressed in first exposure during the time course, we found proteins involved on fatty acids oxidation and energy production. This suggests that, for reasons that need to be further analyzed, ticks which parasite naïve hosts obtain energy from fat to feed, which may explain the slower weight gain that we observed in these ticks (**Supplementary Figure 2**). Cluster 2 represented the cluster with the highest number of DEGs and was constituted by DEGs overexpressed in second exposure at late feeding time points. Here, we found that ticks of second exposure start to overexpress proteins related to the behavior to starvation during the feeding. With the mechanism under this overexpression to be uncovered, starvation has been related to more aggressive conduct towards the infection and feeding from the host (Rosendale et al., 2019), which can explain the faster weight gain and may help the tick to keep feeding on an immunized host. We also found proteins involved in glycosylation pathways. Additionally, the most DEGs in the time course between first and second exposure of the host encode for enzymes in the glycosylation pathways and ribosomal proteins. Since ribosomal components are essential for synthesizing all proteins and glycosylation is responsible for proper function and activity (Mulenga et al., 2013; Vechtova et al., 2018), the results obtained in the time course analysis indicate that ticks do not necessarily rely on genes expressed exclusively against immune hosts. Ticks might instead, increase transcription globally and more rapidly when faced with an immunized host. The differential expression of such general factors in protein expression and function may also enlighten us about the broad range of hosts the tick can parasitize. It also explains the different degrees of tick feeding success and host permissibility (Narasimhan et al., 2019).

Thus, rather than devising a strategy for each host and attempting to match the expansive repertoire of host antibodies, it seems that ticks are using an accelerated, amplified, but imperfect transcriptional program as a general strategy to feed on most but not all immune animals. Therefore, one possibility is that the coding portion of the transcriptome is only partly responsible for host-specific evasion.

Beyond the changes in the expression of coding transcripts, our dataset may also contain information on non-coding RNA/transcripts that we did not study because we based our annotations on proteomics databases. It will be interesting to weigh how specific tick salivary proteins are compared to molecules such as microRNAs. Proteins such as protease inhibitors demonstrate cross-species reactivity (Chmelař et al., 2017), whereas we previously detected several miRNAs predicted to target human immune pathways, but any cross-reactivity remains to be determined (Hackenberg et al., 2017). Therefore, once more information is uncovered on the role of non-coding RNAs in tick feeding and host manipulation (Bensaoud et al., 2020), we may revisit this dataset using established criteria and methodologies (Hackenberg et al., 2017). Consequently, we will determine if tick non-coding RNA activity is altered during the second exposure.

Tick bites can transmit viral or bacterial pathogens that cause severe long-term complications such as paralysis, central nervous system inflammation, cognitive dysfunction in tick-borne encephalitis, paralysis, and arthritis in Lyme disease (Centers for Disease Control and Prevention). Although these diseases can be prevented by targeting the pathogens themselves, an alternative solution is to interrupt tick feeding and pathogen transmission. Our findings have implications for both of these strategies in preventing severe human diseases. *Via* re-exposure of rabbit hosts to ticks, we expected that the products of the most essential, the most differentially regulated transcripts would be promising vaccine antigen candidates, especially for products secreted from the salivary glands (Karim and Ribeiro, 2015). However, the transcripts that we identified are responsible for translation or post-translational modification of proteins. As intracellular enzymes, this makes them poor candidates as vaccine antigens. This finding may explain the lack of success so far in producing an anti-tick vaccine because antibodies cannot directly inhibit protein synthesis.

Furthermore, whether the result is alternative or increased glycosylation, glycans remain less immunogenic than proteinaceous molecules, making them difficult to target. We would discover more promising candidates by correlating the transcriptome with host parameters. Ideally, we can identify specific host molecules (e.g., antibodies) that ticks are reacting to and the related mechanisms. We need to correlate the transcriptome to, at the very least, measurable parameters: host antibody titers, duration of tick attachment, tick engorgement weight, etc. Especially during the second exposure, such comparisons will inform us about the host-parasite interaction and which transcripts help overcome the immune response. In addition to anti-tick therapy, our findings are indirectly relevant for preventing arbodiseases. As pathogens invade both the midgut and the salivary glands of the parasite before being transmitted, activity in both tissues will influence how pathogens are transmitted. The transcriptomes we produced help distinguish the tick’s changes during feeding from those induced by pathogens. However, even independent of the influence of any pathogen, our results indicate that both tissues are most active during the first 48 hours – throughout this period, we measured the most differential gene expression between time points. These changes were more subdued during the second exposure. Our findings support those from Narasimhan et al., who demonstrated that the first 24 hours are crucial to tick feeding success (Narasimhan et al., 2007) – immunity against salivary products produced during this period is crucial to interrupt tick feeding and pathogen transmission. Moreover, in that study, the lower engorgement weight and rejection of almost all ticks within 72 hours of a second exposure also support that an adaptive host immune response is mounted and that ticks react to this secondary response. Finally, our finding that glycosylation is differentially regulated between exposures may indicate that the tick depends on differential glycosylation for host evasion. Any pathogen may also be differentially glycosylated due to secondary infections (Lattová et al., 2020). Therefore, glycan-reactive antibodies, glycoproteins as vaccine antigens, or lectins may prove to be effective for (post-exposure) prophylaxis against the tick and pathogens alike.

Lastly, we provided the research community with a useful tool to browse the results obtained in this study or to find new features for their own sequence. IxoriDB is a database-associated webpage that can be accessed through the link https://arn.ugr.es/IxoriDB/.

## 6 Conclusion

In summary, we designed an experiment that compares the gene expression profiles of ticks fed on naïve or re-exposed immune rabbits. Notably, we sequenced individual rather than pooled specimens, fed related pathogen-free ticks, and produced quality-controlled data supporting sialome switching for successful tick feeding. As the (adaptive) immune response is amplified, accelerated, and specific upon antigen/pathogen reencounter, our data indicate that *I. ricinus* also evades the host immune system *via* altering its transcriptional profile. The transcriptomic changes between ticks exposed to naive and immunized rabbits may be a form of parasite adaptation and a sialome switching itself. By studying the rabbit that permits repeated tick feeding, we determined that the tick reacts to an immune host and adaptive immune response by increased translation and increased or altered post-translational glycosylation. This raises the question of whether these transcriptional changes also allow the tick to overcome an actively immunized/vaccinated host and not only a previously tick-exposed host. Studying the glycobiology of ticks will determine how much this altered activity permits the tick to progress through the different feeding stages and/or evade the host immune response, adding another layer of complexity to proteomics. Only by understanding successful mechanisms of natural parasite evasion or host resistance can we interrupt tick feeding, arbodisease transmission and confer resistance to hosts of arthropod vectors.

## Data availability statement

The datasets presented in this study can be found in online repositories. The names of the repository/repositories and accession number(s) can be found in the article/**Supplementary Material**.

## Ethics statement

All animal experiments were carried out in accordance with the Animal Protection Law of the Czech Republic No. 246/1992 Sb, ethics approval No. 095/2012, and protocols approved by the responsible committee of the Institute of Parasitology, Biology Centre of the Czech Academy of Sciences.

## Author contributions

JMM performed data analysis, web development and drafted the manuscript. CG-M and EA-P performed web development. BC and IM performed all the validation experiments. MAJ, JK and LAM performed tick feeding experiments and sample preparation for the NGS. MH performed data analysis and drafted the paper. MK and CB designed the project and drafted the manuscript. All authors contributed to the article and approved the submitted version.

## Funding

MK received funding recived from the Grant Agency of the Czech Republic (grant19-382 07247S) and ERD Funds, project CePaVip OPVVV (No. 384 CZ.02.1.01/0.0/0.0/16_019/0000759). MH received a grant from Programa Operativo FEDER de Andalucía (A-BIO-481-UGR18). CB received the European Union within ESIF in frame of Operational Programme Research, Development and Education (project no. CZ.02.2.69/0.0/0.0/20_079/0017809).

## Acknowledgments

We would like to thank Dr. Justin Chan for his English corrections and constructive comments on the manuscript.

## Conflict of interest

The authors declare that the research was conducted in the absence of any commercial or financial relationships that could be construed as a potential conflict of interest.

## Publisher’s note

All claims expressed in this article are solely those of the authors and do not necessarily represent those of their affiliated organizations, or those of the publisher, the editors and the reviewers. Any product that may be evaluated in this article, or claim that may be made by its manufacturer, is not guaranteed or endorsed by the publisher.

## Supplementary material

Supplementary Figure 1 Exploratory plots of the transcriptome assembly. **(A)** Principal component analysis of the expression profiles for the samples from *Ixodes ricinus*. Samples from both midgut and salivary glands are represented. Tissue, feeding time point and exposure number are represented by different colours. Additionally, exposure number is also represented by different shapes. The codes for each sample are as follows: tissue (MG or SG), exposure number (unfed, first or second), and feeding time point in hours (12h, 24h, 48h, 72h and 96h). **(B)** Correlation analysis for the samples from *Ixodes ricinus*. The matrix shows the level of correlation of the expression patterns between the 88 samples in this study. Darker blue indicates stronger correlation. The codes for each sample are as follows: tissue (MG or SG), exposure number (unfed, first or second), feeding time point in hours (12h, 24h, 48h, 72h and 96h) and number of biological replicates (R1, R2 or R3).

Supplementary Figure 2 Weight distribution and weight-gain dynamic of *Ixodes ricinus.* **(A)** Distribution of weight among different exposures and feeding time points. **(B)** Dynamic of the weight gain during feeding in the first and second exposure.

Supplementary Figure 3 Distribution of biological process GO terms. Graph showing the distribution of the 3190 GO terms categorized as biological process annotated in the set of unique coding regions. A darker orange color indicates a higher Blast2GO Node Score.

Supplementary Figure 4 Distribution of molecular function GO terms. Graph showing the distribution of the 4362 GO terms categorized as molecular function annotated in the set of unique coding regions. A darker orange color indicates a higher Blast2GO Node Score.

Supplementary Figure 5 Distribution of cellular component GO terms. Graph showing the distribution of the 3335 GO terms categorized as cellular component annotated in the set of unique coding regions. A darker orange color indicates a higher Blast2GO Node Score.

Supplementary Figure 6 Analysis, intersection and functional characterization of the unique coding regions with critical coefficients of variation for midgut and 3 samples from salivary glands. **(A)** Intersection analysis and interaction network for the 1000 unique coding regions with the lowest coefficients of variation for each condition in midgut. **(B)** Intersection analysis and interaction network for the 1000 unique coding regions with the highest coefficients of variation for each condition in midgut. **(C)** Intersection analysis and interaction network for the 1000 unique coding regions with the lowest coefficients of variation for each condition in 3 random samples of salivary glands. **(D)** Intersection analysis and interaction network for the 1000 unique coding regions with the highest coefficients of variation for each condition in 3 random samples of salivary glands.

Supplementary Figure 7 Representation of expected gene expression for protein coding genes from transcriptomic data. The 6 highly expressed genes are represented by their FPKM values at different time points for both salivary glands and midgut after first exposure **(A, B)** and second exposure **(C, D)**.

Supplementary Figure 8 RT-qPCR analyses of transcripts in tick salivary gland and midgut. The relative expression of protein coding genes is represented by the delta Ct values normalized to the average of housekeeping genes (*ef* and *actin*). The data represents the relative expression of salivary gland and midgut extracted from ticks after first exposure **(A, B)** and second exposure **(C, D)**.

Supplementary file 1 Excel file with all the annotation and features of the unique coding regions. This file shows the following information: transcript sequences, CDS sequences, peptide sequences, families, information about the best hits obtained using blast, GO terms and keywords obtained from Uniprot, Interpro domains and GO terms, final set of GO terms obtained after merging GO terms obtained from Uniprot and Blast2GO, SignalP, TMHMM domains, annotation- and secretion-based classes, tissue specificities with the fold change for midguts and salivary glands, information about differential expression (the coding is <tissue>|<exposure number of the first sample>_<time feeding point of the first sample>|<exposure number of the second sample>_<time feeding point of the second sample><UP or DOWN expressed>), fold changes and posterior probabilities of being differentially expressed, and expression for every sample in FPKM.

Supplementary file 2 Excel file with coefficients of variation and stability ranks for *Ixodes ricinus* unique coding regions. Here, the coefficients of variation, stability ranks and their means for every tissue and condition are shown for every unique coding region.

Supplementary file 3 Excel file of DEGs in the time course with the clusters they are associated with for both midgut and salivary glands. Here, two columns are presented, the first one correspond to the ID of the DEG in the time course, and the second one the cluster in which it have been included by the hierarchical clustering process. The first sheet correspond to clusters and DEGs in midgut and the second sheet to clusters and DEGs in salivary glands.

Supplementary file 4 Ontology enrichment analysis for the DEGs in the time course that showed accelerated early overexpression during the second exposure in midgut. Here, five columns are shown, the first, second and third columns correspond to the ID, description and type of the GO term, respectively, the fourth column shows the strength of the enrichment (log10(observed/expected)), and the fifth one the statistical significance of the enrichment in the form of false discovery rate.
