# Supplementary figures and images for: Transcriptomic analysis of the tick midgut and salivary gland responses upon repeated blood-feeding on a vertebrate host
Source: Front Cell Infect Microbiol. 2022 Aug 4;12:919786. doi: 10.3389/fcimb.2022.919786 (PMC9386188; doi:10.3389/fcimb.2022.919786)

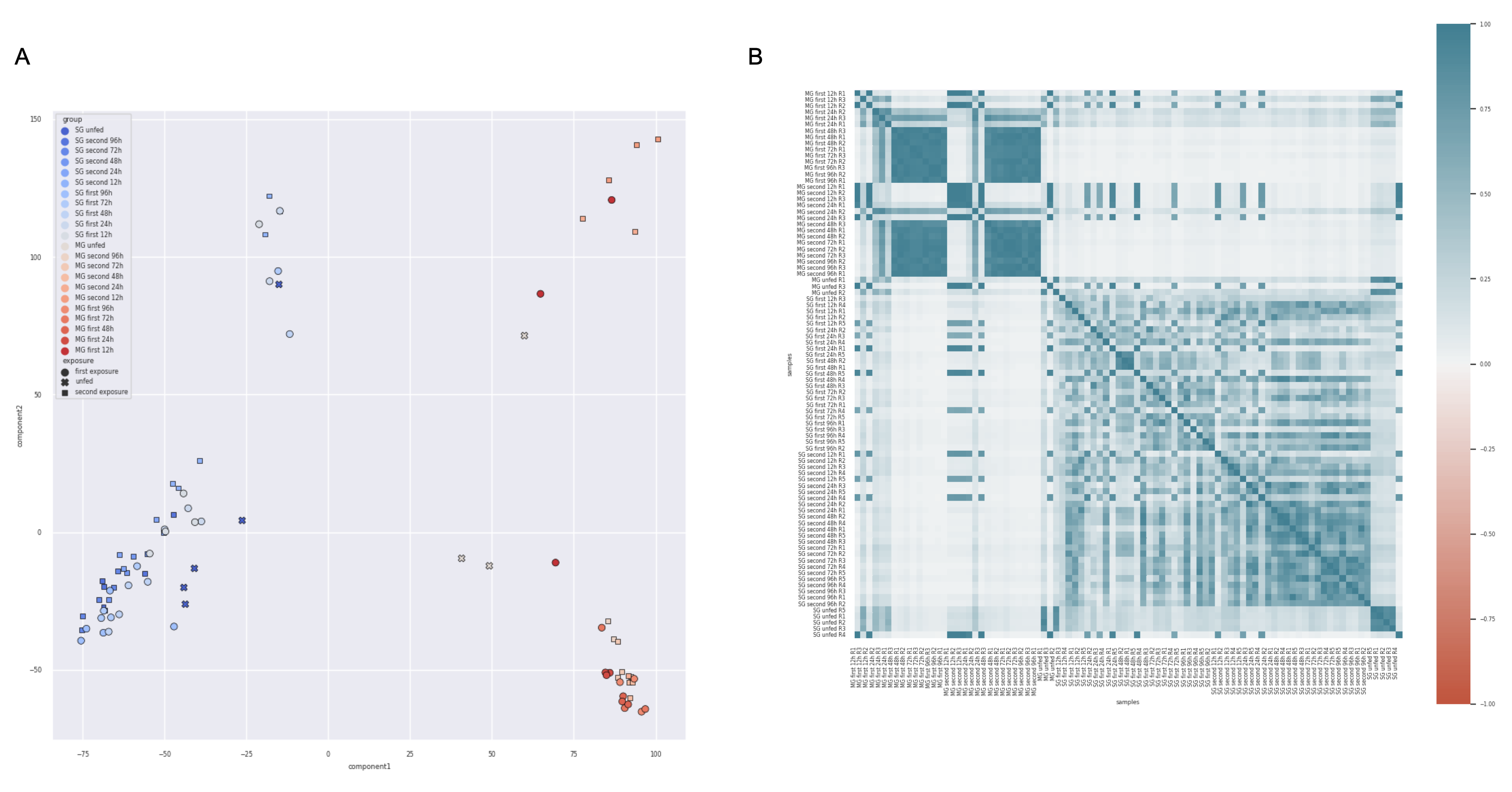

Supplement: Supplementary Figure 1 — Exploratory plots of the transcriptome assembly. (A) Principal component analysis of the expression profiles for the samples from Ixodes ricinus. Samples from both midgut and salivary glands are represented. Tissue, feeding time point and exposure number are represented by different colours. Additionally, exposure number is also represented by different shapes. The codes for each sample are as follows: tissue (MG or SG), exposure number (unfed, first or second), and feeding time point in hours (12h, 24h, 48h, 72h and 96h). (B) Correlation analysis for the samples from Ixodes ricinus. The matrix shows the level of correlation of the expression patterns between the 88 samples in this study. Darker blue indicates stronger correlation. The codes for each sample are as follows: tissue (MG or SG), exposure number (unfed, first or second), feeding time point in hours (12h, 24h, 48h, 72h and 96h) and number of biological replicates (R1, R2 or R3). [file Image_1.png]

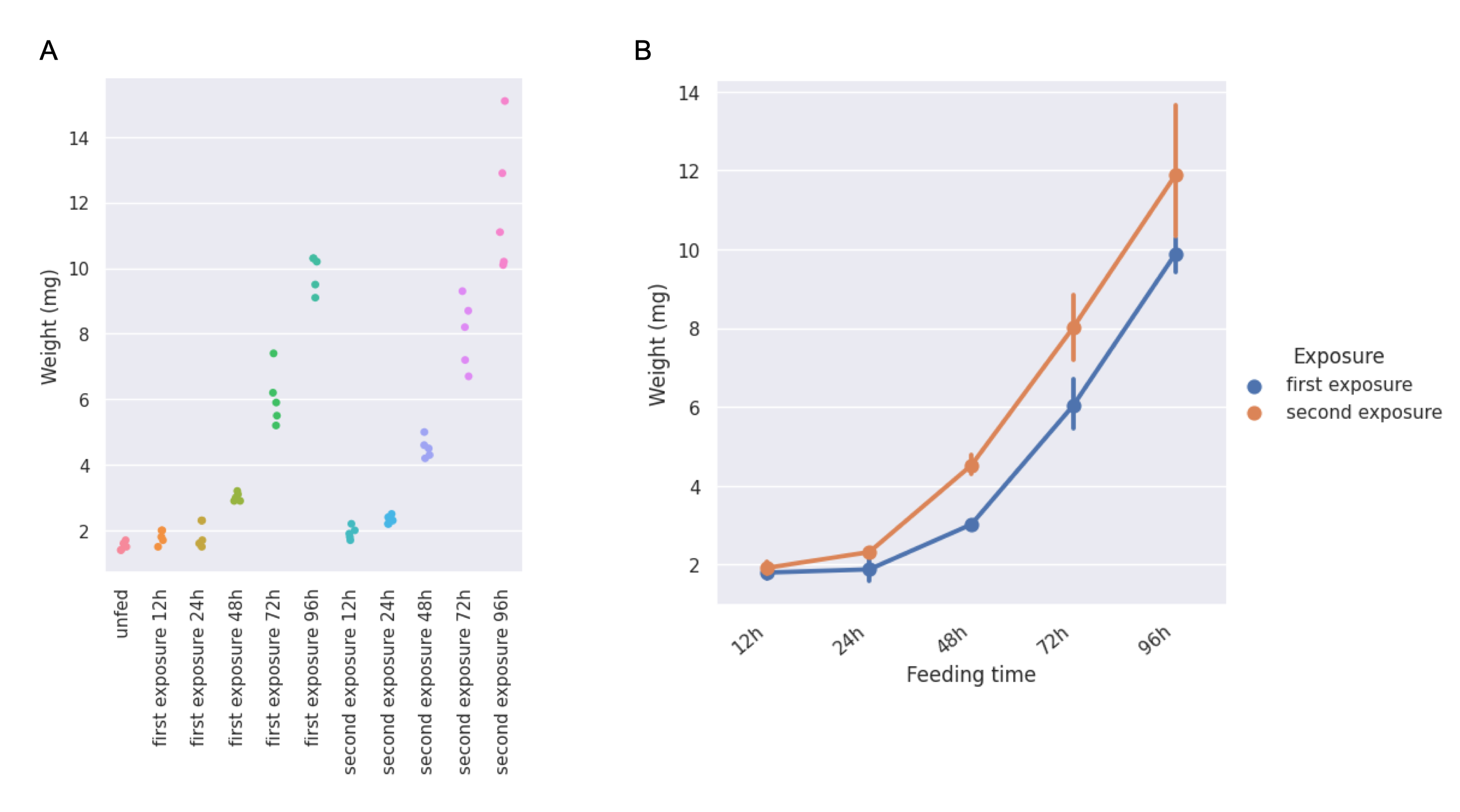

Supplement: Supplementary Figure 2 — Weight distribution and weight-gain dynamic of Ixodes ricinus. (A) Distribution of weight among different exposures and feeding time points. (B) Dynamic of the weight gain during feeding in the first and second exposure. [file Image_2.png]

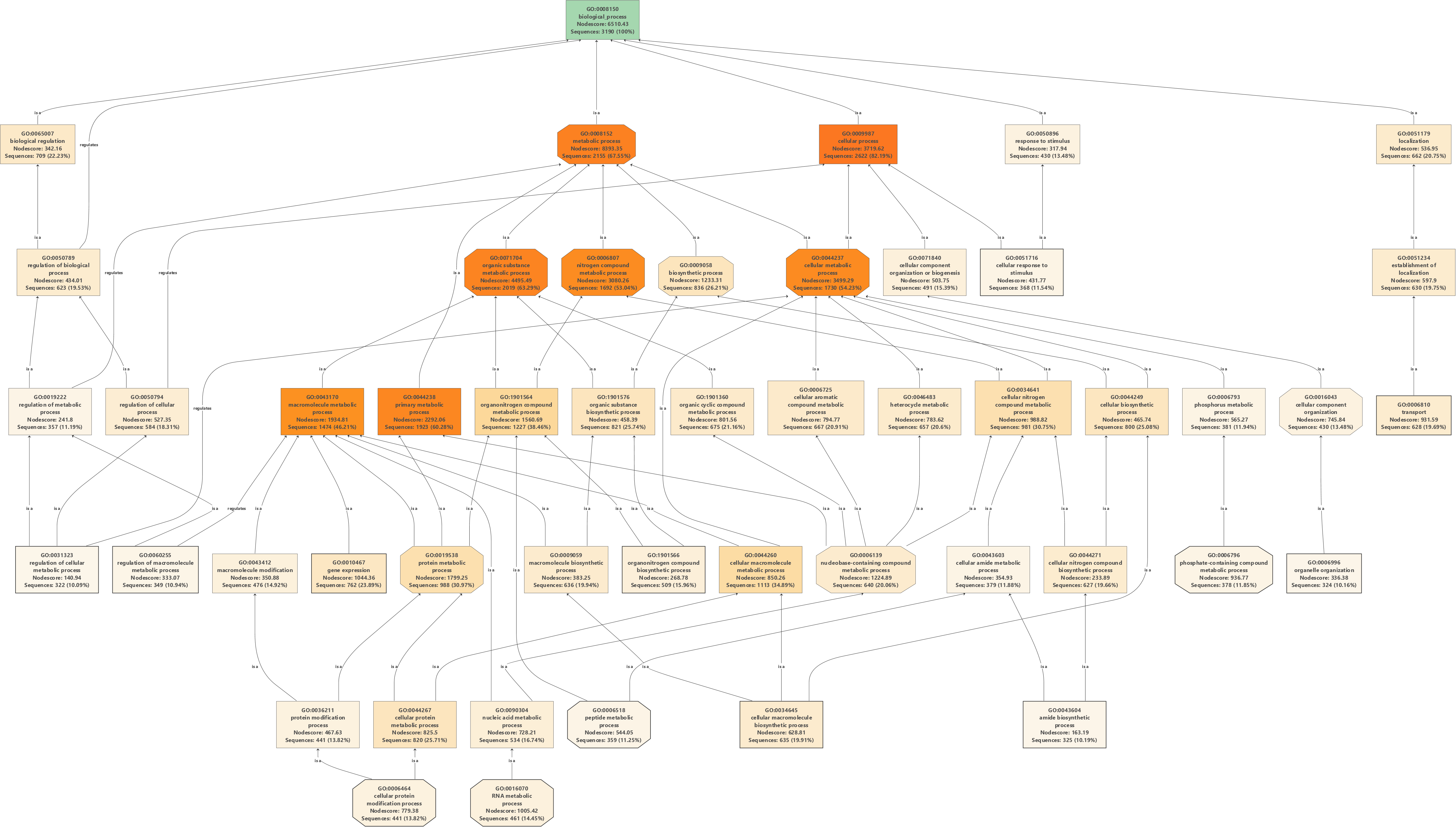

Supplement: Supplementary Figure 3 — Distribution of biological process GO terms. Graph showing the distribution of the 3190 GO terms categorized as biological process annotated in the set of unique coding regions. A darker orange color indicates a higher Blast2GO Node Score. [file Image_3.png]

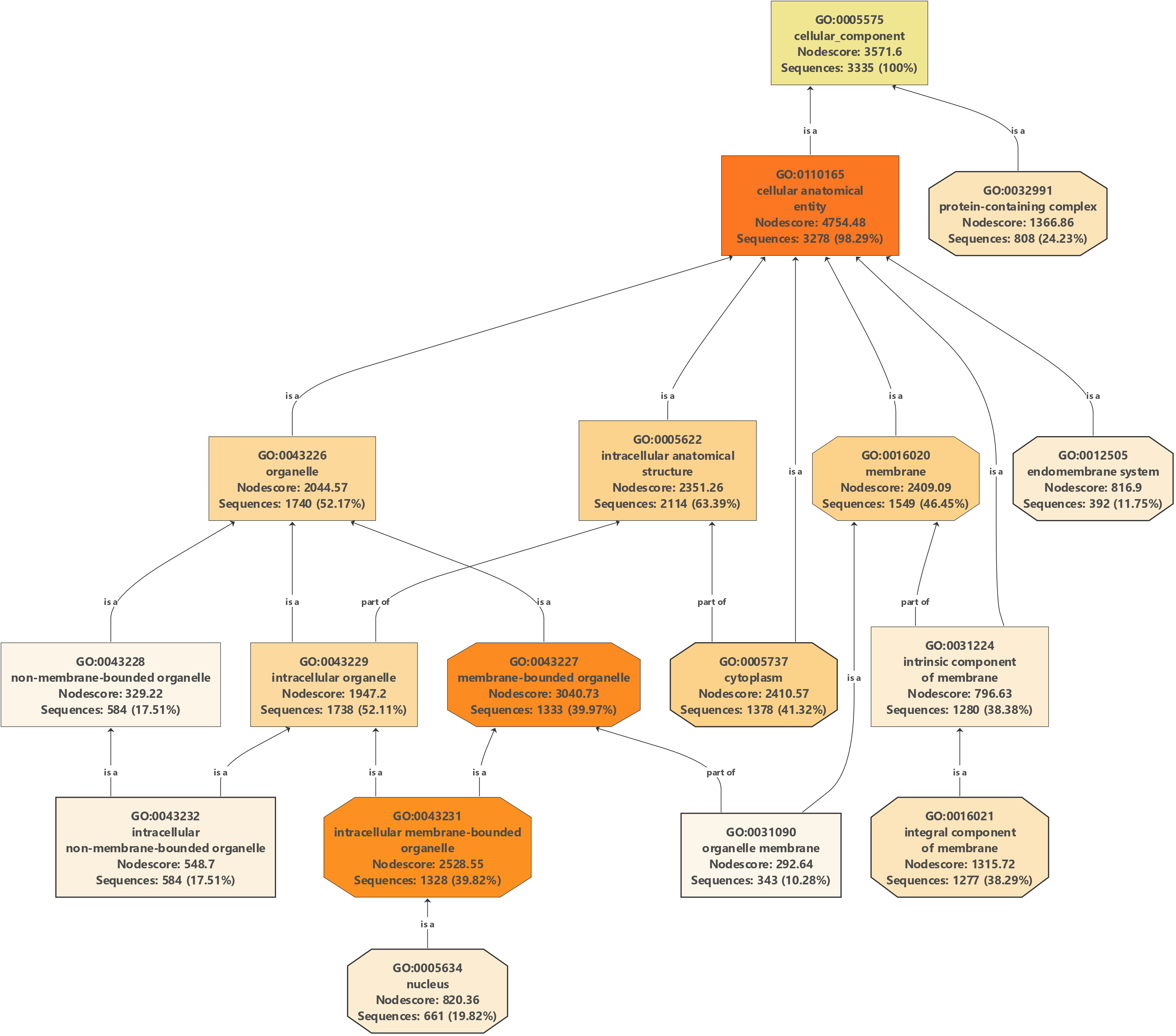

Supplement: Supplementary Figure 4 — Distribution of molecular function GO terms. Graph showing the distribution of the 4362 GO terms categorized as molecular function annotated in the set of unique coding regions. A darker orange color indicates a higher Blast2GO Node Score. [file Image_4.png]

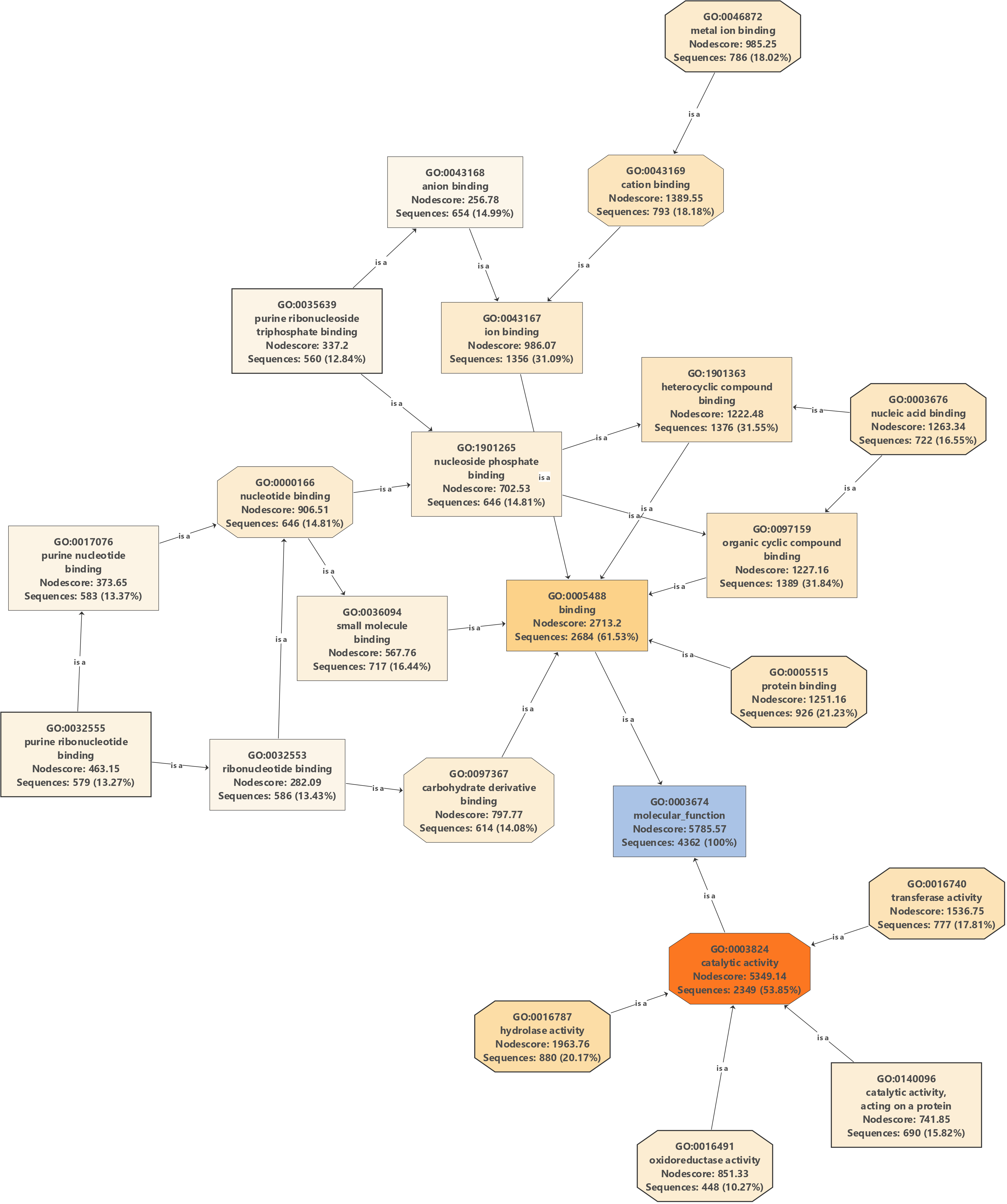

Supplement: Supplementary Figure 5 — Distribution of cellular component GO terms. Graph showing the distribution of the 3335 GO terms categorized as cellular component annotated in the set of unique coding regions. A darker orange color indicates a higher Blast2GO Node Score. [file Image_5.png]

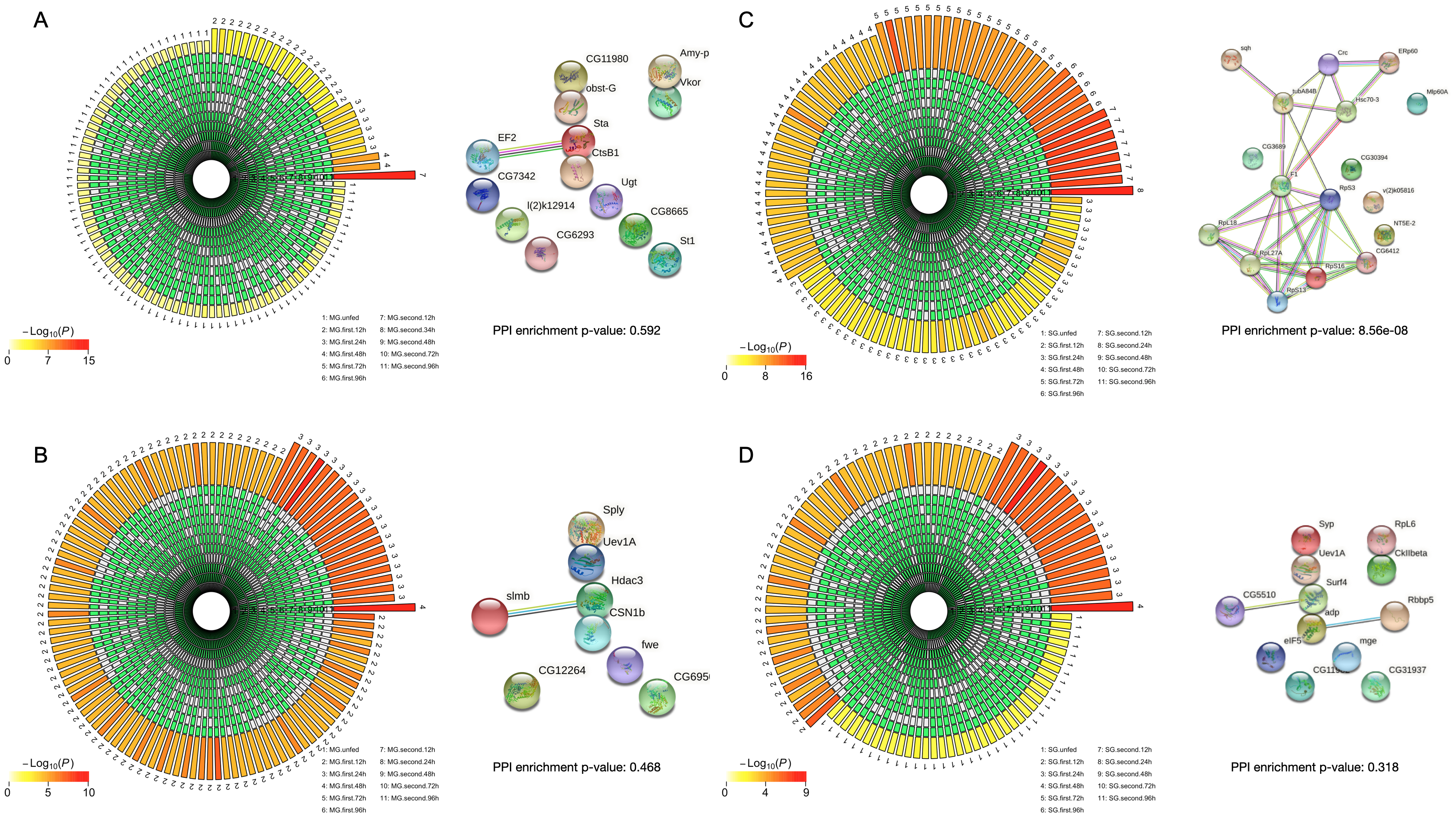

Supplement: Supplementary Figure 6 — Analysis, intersection and functional characterization of the unique coding regions with critical coefficients of variation for midgut and 3 samples from salivary glands. (A) Intersection analysis and interaction network for the 1000 unique coding regions with the lowest coefficients of variation for each condition in midgut. (B) Intersection analysis and interaction network for the 1000 unique coding regions with the highest coefficients of variation for each condition in midgut. (C) Intersection analysis and interaction network for the 1000 unique coding regions with the lowest coefficients of variation for each condition in 3 random samples of salivary glands. (D) Intersection analysis and interaction network for the 1000 unique coding regions with the highest coefficients of variation for each condition in 3 random samples of salivary glands. [file Image_6.png]

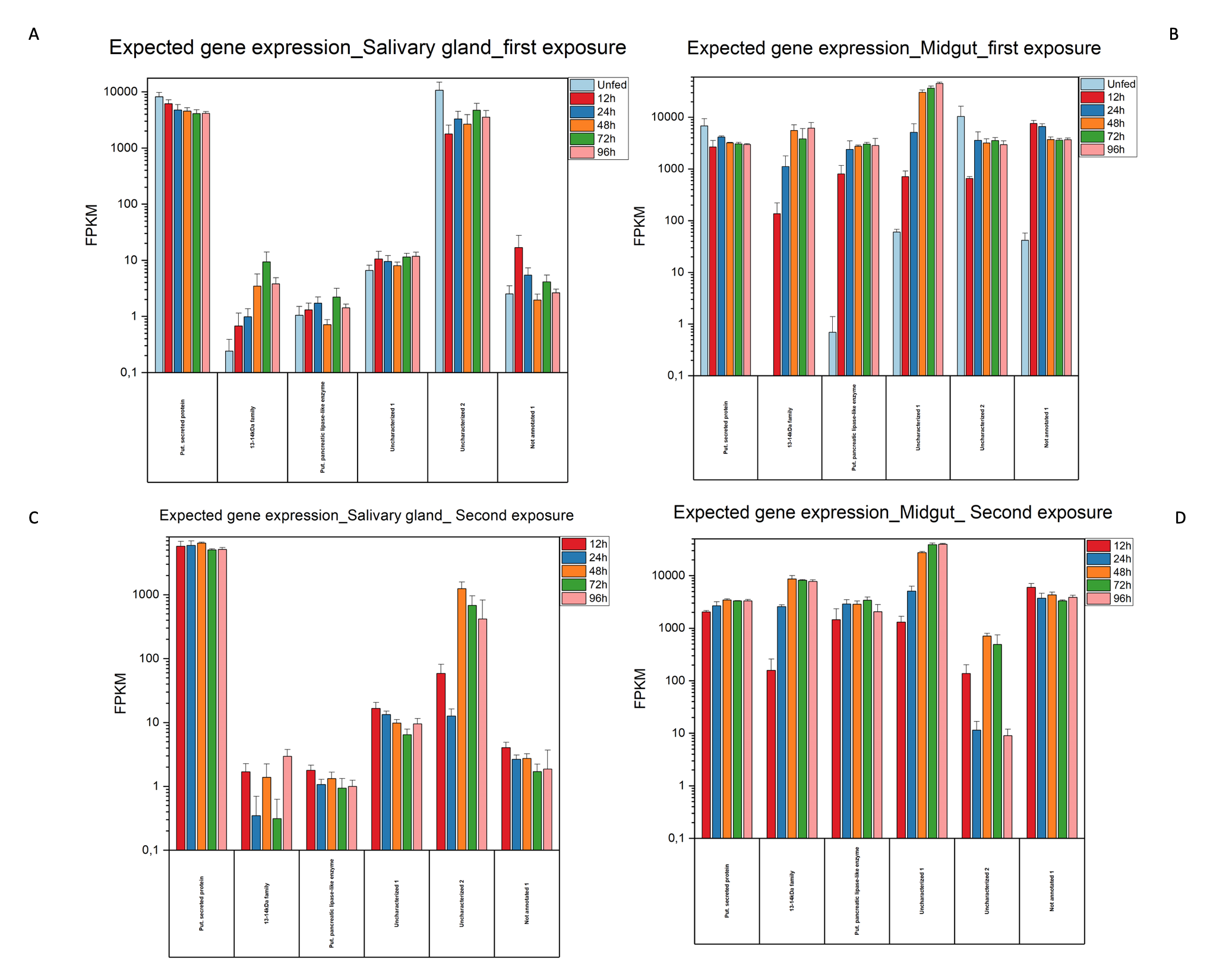

Supplement: Supplementary Figure 7 — Representation of expected gene expression for protein coding genes from transcriptomic data. The 6 highly expressed genes are represented by their FPKM values at different time points for both salivary glands and midgut after first exposure (A, B) and second exposure (C, D). [file Image_7.png]

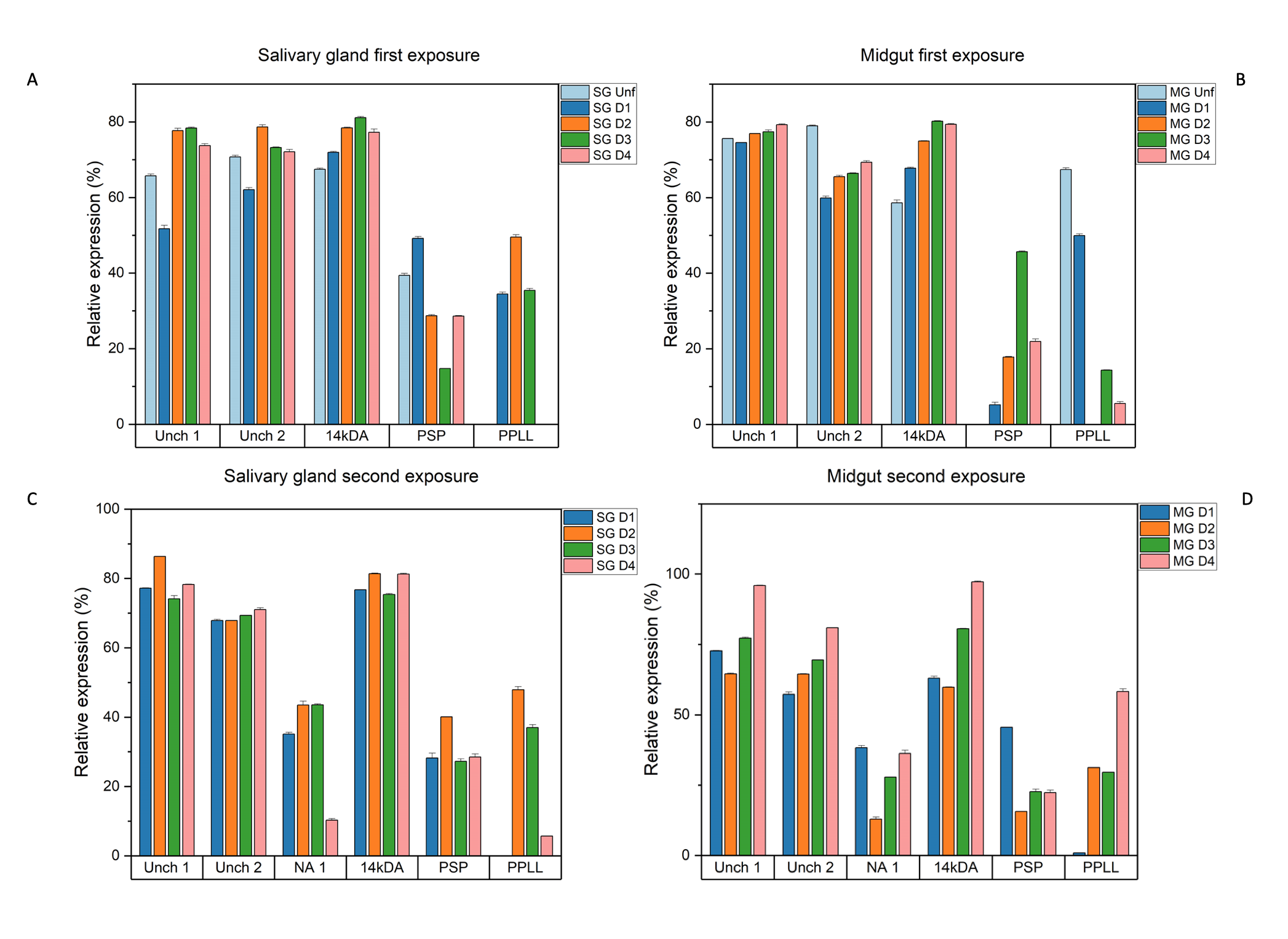

Supplement: Supplementary Figure 8 — RT-qPCR analyses of transcripts in tick salivary gland and midgut. The relative expression of protein coding genes is represented by the delta Ct values normalized to the average of housekeeping genes (ef and actin). The data represents the relative expression of salivary gland and midgut extracted from ticks after first exposure (A, B) and second exposure (C, D). [file Image_8.png]
